# CXCR4 and CXCR6 dually limit T cell entry into the polyomavirus-infected brain

**DOI:** 10.1186/s12974-025-03496-2

**Published:** 2025-06-28

**Authors:** Kalynn M. Alexander, Elia Afanasiev, Arrienne B. Butic, Ge Jin, Mofida Abdelmageed, Anirban Paul, Jo Anne Stratton, Aron E. Lukacher, Samantha A. Spencer

**Affiliations:** 1 Department of Cell and Biological Systems, Pennsylvania State University College of Medicine, Hershey, PA 17033 USA; 2 Department of Neurology and Neurosurgery, Montreal Neurological Institute, McGill University, Montreal, QC Canada

**Keywords:** CXCR4, CXCR6, Ependyma, MuPyV, T cells

## Abstract

T cell responses are vital for controlling viral infection in the central nervous system (CNS), but must be tightly regulated to limit tissue-damaging inflammation. Using mouse polyomavirus (MuPyV) CNS infection, an in vivo model for JCPyV-induced Progressive Multifocal Leukoencephalopathy (PML), we investigated sites of early infection, immune responses, and recruitment of T cells to the brain. Multiplexed error-robust fluorescence in situ hybridization (MERFISH) single-cell spatial transcriptomics was applied to assess the regionality of virus infection and brain-resident cell and infiltrating leukocyte responses. MERFISH, immunofluorescence microscopy, quantitative PCR, and flow cytometry demonstrate that the ependyma is the predominant region of MuPyV CNS infection and localization of T cells, and implicated CXCR4 and CXCR6 in T cell migration to the ependyma and subventricular zone. Using CXCR6 knockout mice and a specific CXCR4 small molecule antagonist, we found that the combined impairment of CXCR6 and CXCR4 signaling resulted in elevated infiltration of T cells in the MuPyV-infected brain. This work demonstrates that CXCR4 and CXCR6 act in a nonredundant fashion to restrict T cell accumulation to the polyomavirus-infected ependyma, with important implications for ongoing efforts to use JCPyV-specific T cell adoptive immunotherapy for PML.

**Supplementary Information:**

The online version contains supplementary material available at 10.1186/s12974-025-03496-2.

## Background

The CNS has historically been considered immune-privileged, where entry of immune cells from the blood is blocked by endothelial cell barriers and from the cerebrospinal fluid (CSF) by tight junctions of the ventricular ependyma. Accumulating literature, however, documents the presence of memory T cells in the brain parenchyma of humans dying from non-neurological causes and healthy mice infected peripherally by non-neurotropic viruses [1–3]. By encamping in the CNS, T cells survey the brain to clear microbial infections, but can incur bystander tissue damage directly or via instigation of inflammatory responses. Tight regulation of T cell entry into the CNS is essential to balancing pathogen control against neuropathology [4, 5].

JCPyV, a ubiquitous human-specific virus, hides lifelong in the urinary tract of healthy individuals, but under certain conditions of inherited or acquired immunocompromise neurovirulent variants can emerge and cause PML [6]. CD8 T cells localize to demyelinating lesions in brains of PML patients, and PD-1 blockade and JCPyV-specific CD8 T cell adoptive immunotherapies have found success in treating this high morbidity/morality disease [7, 8]. Whether JCPyV invades the brain from the blood via the blood-brain barrier (BBB) or into the CSF via the blood-choroid plexus route [i.e., the blood-CSF barrier (BCSF)] is unclear. Using monolayers of choroid plexus epithelial cells and brain endothelial cells in transwell cultures, Atwood and coworkers found that JCPyV preferentially enters the brain via the BCSF [9, 10]. Where the virus then gains access to the brain parenchyma is unknown.

The MuPyV CNS infection model has proven valuable in garnering insights into early events in JCPyV entry into the brain and the anti-viral T cell response [11]. Among these findings is the ependyma as the major site of MuPyV infection and immune cell recruitment [5, 12, 13]. The ciliated single cell ependyma layer, the sole physical barrier between the CSF and the brain parenchyma, is contiguous with the choroid plexus epithelium that supports productive JCPyV replication and is likely to shed virus into the CSF [14]. The MuPyV infection model, then, supports the scenario for JCPyV to subsequently infect the ependyma. Moreover, astrocytic end feet abut the ependyma, suggesting a plausible direct transit of JCPyV to astrocytes. Release of infectious virus from these periventricular astrocytes may constitute an initial step of JCPyV invasion into the brain parenchyma. Given its intimate proximity to the subventricular zone (SVZ) neurogenic niche, it is noteworthy that JCPyV infects oligodendrocyte precursor cells (OPCs) and perturbs their differentiation [15]. In this connection, we found MuPyV-infected astrocytes in the periventricular region [16]. We previously described the localization of virus-specific T cells at the ependyma during acute MuPyV CNS infection. How T cells are guided to the infected ependyma is an important question as the site where they mount frontline defense of the brain parenchyma.

Compared to astrocytes and microglia, the nature of ependymal cell responses to CNS infection is underappreciated. A few viruses have been reported to infect the ependyma in mouse models (e.g., influenza, mumps, T1 reovirus, lymphocytic choriomeningitis virus, and Theiler’s murine encephalomyelitis virus) [17–20]. In humans, ependymal cells and choroid plexus epithelial cells express the ACE2 receptor for SARS-CoV-2 binding, implicating virus-induced effects on BCSF barrier integrity in COVID-19 neurological deficits [18, 19, 21].

Applying single-cell resolution spatial transcriptomics to understand the interactions between MuPyV with ependyma and T cells, we uncovered chemokine receptors and ligands responsible for their homing [22]. By immunofluorescence microscopy, flow cytometry, knockout mice, and selective small molecules inhibitors, we determined that two chemokine receptor-chemokines axes, CXCR4-CXCL12 and CXCR6-CXCL16, act nonredundantly to limit entry of virus-specific T cells to the periventricular region during acute MuPyV CNS infection. The involvement of two chemokine receptors indicates the need for careful control of antiviral T cell responses in the infected CNS to balance tissue defense and damage.

## Methods and materials

### Mice

The C57BL/6 female mice were purchased from the Jackson Laboratories. B6.129P2-Cxcr6^tm1Litt^/J (Cxcr6^−/−^), B6;129-Gt(ROSA)26Sor^tm1(cre/ERT)Nat^/J (ROSA-Cre^ERT^), and B6.129P2-Cxcr4^tm2Yzo^/J (Cxcr4 ^fl/fl^) mice were obtained from Jackson Laboratories and bred in our animal facilities to generate ROSA-Cre^ERT^ x Cxcr4^fl/fl^ mice (referenced as Cre+ or Cre-). Mice were used at 8–10 weeks of age. The animals were kept at a constant temperature of 28 °C with a 12-hour light/dark cycle and were fed and watered ad libitum. All mice were housed at the Pennsylvania State University College of Medicine, monitored and cared for by the Department of Comparative Medicine. All B6 wild type mice purchased from the Jackson Laboratory were female. In experiments using other strains, both male and female mice were used.

### Mouse polyomavirus infection

Mice were anesthetized with ketamine/xylazine and inoculated intracranially (i.c.) with 1 × 10^7^ PFU/mL MuPyV (A2 strain) in 30 ul DMEM. The syringe needle was placed between the eyes and ears of the mouse, adjacent to the midline on the right; the approximate stereotactic location was + 0.3 mm Anterior, -1.0 mm Lateral (Right), and − 3.0 mm Ventral. Sham mice were injected with the equivalent volume of DMEM. Mice were euthanized at 4, 8, 30, and 50 days post infection (dpi).

### Tissue preparation for MERFISH Spatial transcriptomics

Mice were euthanized at 8 dpi with isoflurane and brains were immediately collected without perfusion. Brains were cut at the midline; the contralateral hemisphere (with respect to the inoculation site) was from the infected mouse and the ipsilateral hemisphere from the sham mouse. Both hemispheres were frozen in OCT on dry ice and transferred to a -80 °C freezer for storage. Mice were not perfused (either with fixative or heparin PBS) for the MERFISH experiments per manufacturer (Vizgen, Inc.), who recommended immediate placement on ice to prevent RNA degradation, particularly as brain sections from two mice (infected vs. sham) were used per block. Fresh frozen tissue was recommended as being preferable for RNA analysis rather than FFPE tissue.

Prior to cutting tissue, the OCT blocks were allowed to warm to -20 °C in the cryostat. The tissue was cut in 10 μm sections for slides horizontally from a depth of 3–4 μm to include the lateral ventricles and choroid plexus. Several sections of tissue from the same region were also collected and processed for checking RNA quality. Slide tissue was fixed with 4% PFA, washed three times with 1x PBS, and stored in 70% ethanol until processing. Sections for RNA quality were purified using Trizol and subsequent phenol/chloroform extraction, then resuspended in 20 µL RNAse-free water. RNA samples were run on the Agilent BioAnalyzer NanoChip by the Genome Sciences Core Facility of the Penn State College of Medicine. Samples for the MERFISH spatial transcriptomics runs had RNA integrity scores of 8.6 and 8.8.

### MERFISH spatial transcriptomics bioinformatics analysis

The MERSCOPE instrument and attached computer were used for preliminary processing, providing a detected transcripts file indicating the location in micron space of each detected transcript [23]. Transcripts were then assigned to 8 by 8 micron pseudospots using the functionality provided by the UMAT tool (available at https://github.com/namemcguffin/umat*)*, without flattening (meaning spots were generated separately for each of the 7 z-stacks), saving generated spot by gene matrices with associated spatial metadata as an AnnData object on disk in the h5ad format [24].

The AnnData objects generated by the UMAT tool were then converted into Seurat objects in R using the reticulate library to convert between Python and R matrix/table formats [24–26]. Resulting individual sample objects were first filtered to remove low transcript spots (below 8 transcripts per), the log-normalized expression values for each were calculated using Seurat’s NormalizeData function, and then they were merged into a single object [25]. BANKSY was used to enrich spots with information from neighbouring spots, with the aim of improving clustering results (lambda parameter set to 0.2) [27]. BANKSY was run using the RunBanksy function from the SeuratWrappers library, with a custom modification applied to the SeuratWrappers::get_locs function (via R’s assignInNamespace functionality) to resolve an issue with incorrect fetching of z-coordinates. Low expression spots (below 70 counts in the BANKSY assay) were then filtered out. Following this, principal components, shared nearest neighbour graph (using the first 15 PCs), and leiden clustering results were calculated using Seurat’s RunPCA, FindNeighbours, and FindClusters functions [25, 28].

Following identification of cluster identities, MERSCOPE counts were pseudo-bulked by sample (*n* = 2 for each condition) for each identified cell type, and differentially expressed genes between the sham and infected conditions were calculated using the glmGamPoi library [29].

### Dissection of the periventricular region

**Fig. 1:**
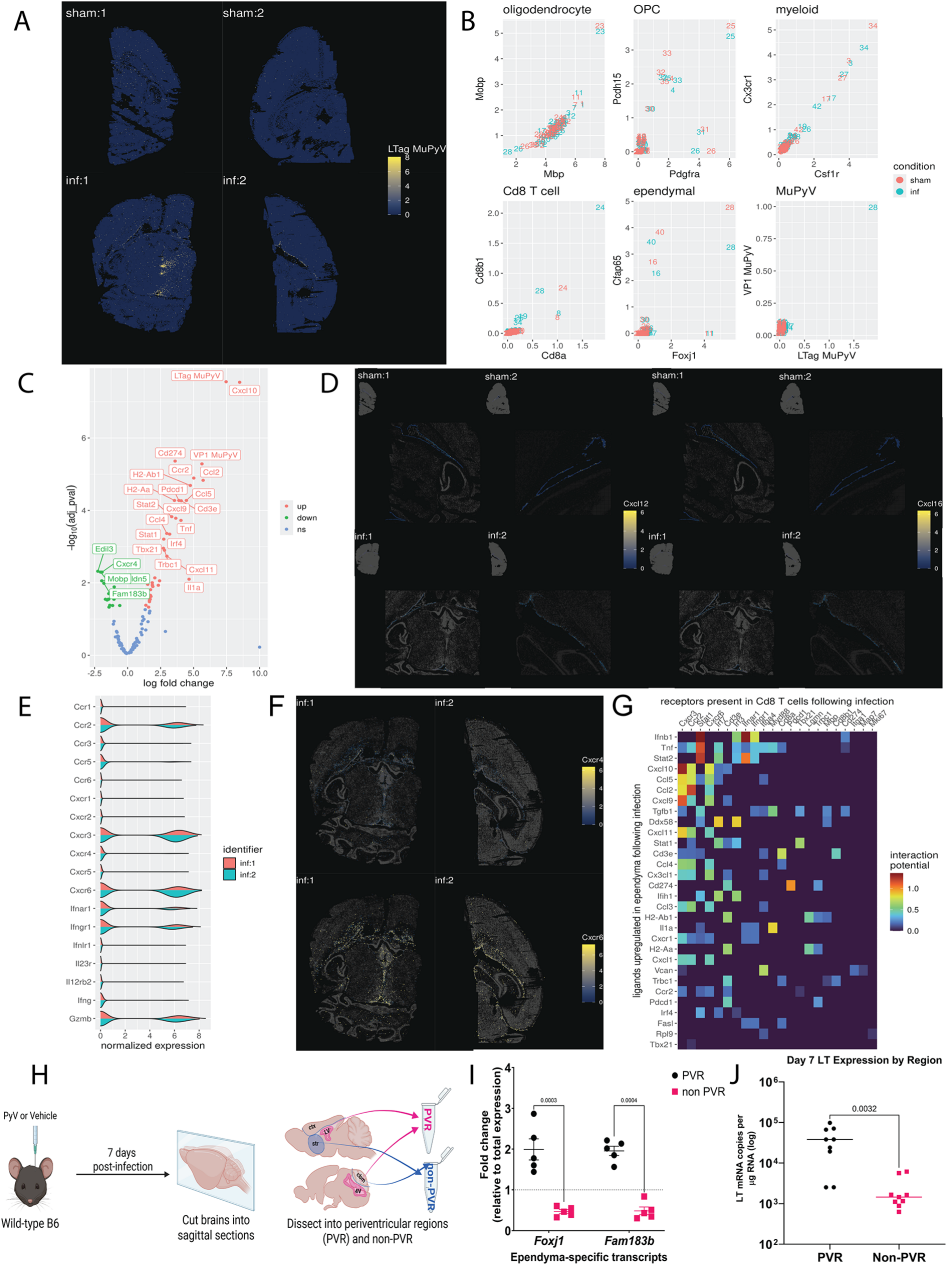
Spatial transcriptomics reveals virus-induced changes in gene expression in the ependyma as well as the primary location of infection in situ. In two independent experiments, wild-type (WT) mice were injected i.c. with vehicle (sham) or MuPyV (infected), and brains collected at 8 dpi. **A**: Expression of LTag transcripts in each spot in sham and infected samples. Spots are placed according to their x and y coordinates, ignoring their z coordinate (i.e., flattening). Spots are colored according to log-normalized LTag transcript expression. **B**: Expression of cell markers in specific clusters. Different clusters are placed according to their average log-normalized expression of different genes on the x and y axes. Values were computed separately for the sham and infected conditions, as represented by color. **C**: Volcano plot displaying DEG analysis for the ependyma. Each gene is represented by a labeled point, organized along the x-axis according to computed log-fold change and by the negative log of the adjusted *p* value on the y-axis. Points are colored according to the nature of the dysregulation (up, down, or not significant). **D**: Expression of Cxcl12 and Cxcl16 transcripts in the ependyma in sham and infected samples. Spots are placed according to their x and y coordinates, ignoring their z coordinate (i.e., flattening). Ependymal spots are colored according to log-normalized Cxcl12 and Cxcl16 transcript expression, with non-ependymal spots greyed out and made semi-transparent. A small overview of the entire section is visible on the top left to better place the location of the ependyma within the tissue. **E**: Density plots of various T cell markers in CD8^+^ T cells in the infected condition. The x-axis represents the level of log-normalized marker expression, y-axis represents the of the plot represents the amount of spots in the dataset with that level of marker expression, and the color represents each individual sample. **F**: Expression of Cxcr4 and Cxcr6 transcripts in Cd8 T cells in infected samples. Spots are placed according to their x and y coordinates, ignoring their z coordinate (i.e., flattening). CD8 T cell spots are colored according to log-normalized Cxcr4 and Cxcr6 transcript expression, with non-CD8 T cell spots greyed out and made semi-transparent. **G**: Interaction potential heatmap between ependymal ligands and T cell receptors. The x-axis plots receptors that were present in over 10% of CD8^+^ T cell spots in the infected condition, the y-axis represents ligands that were found to be significantly upregulated following infection in ependymal cells, and tiles are colored according to interaction potential as established by Browaeys et al. [31] **H**,** I.** The periventricular region of the brain was dissected out and its ependymal gene expression (Foxj1 and Fam183b) confirmed. Diagram made using BioRender.com. The *p*-values shown are matched 2-way ANOVA with post-hoc. **J**: Virus levels are higher in this area compared to the rest of the brain at 7 dpi. Data analyzed by a two-tailed Student’s *t* test

C57BL/6J mice were inoculated or sham-injected i.c., then euthanized with isoflurane at 7 dpi. Brains were removed, cut into 2 mm sections using a sagittal brain matrix, and placed into chilled Hank’s balanced salt solution (Gibco). Each brain was dissected using sterile micro-dissecting scissors and tweezers under a dissecting microscope into the periventricular region (PVR), which included the lateral ventricles, the fourth ventricle, and choroid plexuses, and samples of non-PVR (medial cortex, caudate putamen, and cerebellum). A diagram of the dissected regions is shown in Fig. 1H Each brain’s PVR and non-PVR were then homogenized in 1 mL Trizol before undergoing phenol/chloroform extraction for RNA. The RNA was subsequently used for RT qPCR.

### qPCR

Tissue for RNA, unless otherwise stated, was collected from isoflurane-euthanized mice as a 2 mm coronal section from bregma 0 to -2 contralateral to the injection site for brains, a 2 mm^2^ piece of spleen, or the cervical lymph nodes from one side of the mouse. Tissue was placed in round-bottom tubes with a glass bead and 500 µL-1 mL Trizol and homogenized using a TissueLyser II (Qiagen). RNA was purified using chloroform/phenol extraction and isopropanol precipitation and resuspended in 100 µL RNase-free water. RNA samples were quantified using a NanoDrop spectrophotometer.

Each sample was used to prepare cDNA. To measure viral copies, cDNA was used in a TaqMan (PerfeCTa FastMix II ROX Quantabio) qPCR reaction with primers for large T (LT) antigen (LT; F: AGGAATTGAACAGTCTCTGGG, R: GTCATCGTGTAGTGGACTGTG, Probe: with 5’ 6-FAM/ZEN/3’IABkFQ dye and quencher), and TATA-binding protein (TBP; F: TGTATCTACCGTGAATCTTGGC, R: CCAGAACTGAAAATCAACGCAG, Probe: /56-FAM/ACTTGACCT/ZEN/AAAGACCATTGCACTTCGT/3IABkFQ/). Samples were loaded with a standard curve of LT copies. Plates were run using an Applied Bio Step One Plus qPCR at 95 °C for 5 min, then 40 cycles of 15 s at 95 °C and 1 min at 60 C. LT copies per sample were calculated using Excel.

To measure relative expression of ependyma transcripts, the cDNA, as well as no-reverse transcriptase control cDNA, were used in SYBR green (PerfeCTa SYBR Green FastMix, ROX Quantabio) qPCR reactions. Primers for Foxj1 (F: CTT CTG CTA CTT CCG CCA TGC, R: TCC TCC TGG GTC AGC AGT AAG G) and Fam183b (F: ATC TTG CGG GAA CTC TTT C, R: AGA GTG ATG TCG TGG TAG AC) were used with sham PVR and non-PVR tissue in the following protocol on the Applied Bio Step One Plus qPCR: Control primers for Gapdh (F: TCACCACCATGGAGAAGGC, R: GCTAAGCAGTTGGTGGTGCA), 18 S rRNA (F: CAC TTT TGG GGC CTT CGT GT, R: AGG CCC AGA GAC TCA TTT CTT) and Polb (F: GGT ACT TGG CTA TCA CAG ATG C, R: GGA CAT GCT CGT GGA ACT C) were used as housekeeping genes. To determine the relative expression, the geometric mean of the Ct values of the three housekeeping genes was calculated for each sample, then used with the Ct values of Foxj1 or Fam183b in the ΔΔCt method. The expression of each sample was calculated relative to the combined ΔCt values for the PVR and non-PVR as fold change.

### Tamoxifen treatment of ROSA-Cre^ERT^ x Cxcr4^fl/fl^ mice

ROSA-Cre^ERT^ x Cxcr4^fl/fl^ mice included Cre + and Cre- mice, genotyped per Jackson Laboratories’ instructions. All mice were given 2 mg tamoxifen in 10% ethanol, corn oil i.p., daily for 5 days. One day after the final tamoxifen injection, mice were injected i.c. with MuPyV.

### Alzet osmotic pump delivery of AMD3100

Mice (C57BL/6J or Cxcr6^−/−^, aged 6–8 weeks) were anesthetized with ketamine/xylazine, shaved dorsally, and injected i.c. with MuPyV, then subcutaneously implanted with osmotic pumps (Alzet #1002) with a flow rate of 0.25 µL/hour were filled with either sterile 1x PBS or AMD3100 (plerixafor octahydrochloride, CAS No.: 155148-31-5, MedChemExpress Cat. No.: HY-50912) dissolved in 1x PBS and sterile filtered with a 25 mm PES filter. The AMD3100 concentration was calculated for release at a constant dose of 10 mg/kg/day. The osmotic pumps were inserted subcutaneously at the midline posterior to the scapula, and the incision was closed with dissolvable sutures. Mice were weighed daily and given carprofen daily as needed for pain management. Mice were euthanized at 8 dpi and tissues processed for flow cytometry and RNA extraction. Additional mice underwent cardiac perfusion fixation and processing for immunofluorescence. Pumps were removed, and the remaining volume in each was measured to ensure that each pump was empty.

### Flow cytometry

For experiments requiring intravenous (IV) labeling, mice were injected retro-orbitally with 3 µg antibody [CD45-FITC or CD45-PerCPCy5.5 (BB700 channel)] in 1x PBS for 3 min before euthanasia. Submandibular bleeds were used to collect blood samples prior to euthanasia. All mice were sacrificed and tissues collected (brains, spleens, and/or cervical lymph nodes). Cervical lymph nodes were dissociated through 70 μm filters. Spleens were dissociated through 70 μm filters, then red blood cells were lysed with ACK lysis buffer. Brains were minced and digested with collagenase type I and DNAse I for 20 min at 37 °C. Digested brains were dissociated through 70 μm filters, and a 44–66% Percoll gradient was used to isolate the lymphocytes and remove myelin.

For cell counting, cells were directly added to 2% PFA and mixed with counting beads (Bangs Laboratories, Inc. Cat#580). Suspended cells were stained on 96-well round-bottom plates. For most panels, cells were stained with 1:1000 fixable viability dye (ThermoFisher Fixable Viability Dye eFluor 780, Cat# 65-0865-14, 1:1000) and 1:200 Fc block in 1x PBS at 4 °C for 20 min. Antibodies and their dilutions in FACS buffer are listed in Supplemental Table 1; staining was done at 4 °C for 45 min. For all experiments, cells were then fixed in 2% PFA at room temperature for 15 min. Samples were run on either the Symphony 17 or 23 (BD Biosciences) flow cytometer instrument in Penn State College of Medicine’s Flow Cytometry Core and analyzed using FlowJo software. For experiments utilizing CXCR6-/- mice, which express GFP, FITC antibodies were used in the FMO and compensation control setups and no other markers were labeled with FITC. All gates were drawn based on FMOs. The fold change of cell counts was calculated relative to the average of the control condition within an experiment to remove variation between experiments.

### Immunofluorescence (IF) microscopy

Mice used for immunofluorescence were anesthetized with ketamine/xylazine, then cardiac perfused with 10% heparin in 1x PBS, followed by 10% normal buffered formalin (NBF). The heads were stored in 10% NBF overnight, then the brains were removed and placed into cartridges in 70% ethanol. Paraffin embedding and slide preparation were done by the Department of Comparative Medicine at Penn State University College of Medicine.

Slides underwent antigen retrieval with sodium citrate buffer at pH 6 and permeabilization with 1% Triton X in 1x PBS, then blocking with 5% BSA or 5% donkey serum in PBST (1x PBS with 0.1% Triton-X and 0.05% Tween-20). Slides were stained overnight with primary antibody in 5% BSA in PBST (Supp. Table 1), then stained with secondary antibody for two hours before mounting with ProLong Gold mounting media with DAPI. Slides were imaged using a fluorescent microscope (Leica DM4000) and processed using ImageJ software.

### Statistics

Statistical results were calculated for non-MERFISH experiments using GraphPad Prism Software. For experiments with a single variable, one or two-sample Student’s t test were used. One-sample t tests were done for cell-type chemokine expression to compare to the hypothetical population value of no expression. Two-way, two-sample t tests were performed after assessing normality using the D’Agnostino-Pearson test. For experiments with two variables, repeated measures two-way ANOVA tests were used, as data often represented multiple measurements from a single mouse. Post hoc calculations used the Tukey test, and either compared means only between columns to avoid inter-tissue comparisons, or between columns or rows. The *p*-values for each statistical test are included on each graph and further statistical information is provided in Supplemental Table 3. Mice were randomly assigned to each experimental group.

## Results

### MERFISH spatial transcriptomics analysis of brains during acute mupyv infection

Previous findings identified the ependyma as the dominant site of infection and T cell localization in the brain during acute MuPyV CNS infection [16]. This tight regionality led us to apply spatial transcriptomics to explore the host immune response at this site [5]. Adult mice were injected i.c. with MuPyV or vehicle (“sham”); brains were removed eight days later and sectioned dorsally. For each or two independent experiments, anatomically matched brain sections from an infected mouse and a sham mouse were placed side-by-side on slides and loaded onto MERFISH instrument to probe for a set of 141 transcripts identifying resident/infiltrating cells and inflammation-associated mRNAs (Supp. Table 2). Due to issues with cell segmentation of densely packed cells, MERFISH detected transcripts were binned into 8 by 8 micron “pseudospots” instead of being assigned to cells as determined by an image-based segmentation method. Following data processing, we were able to observe a clear difference between levels of viral LT antigen (LTag) transcripts between the sham and infected conditions (Fig. 1A), with a visibly greater transcript density in the infected condition along the ventricular wall and in the periventricular area. Using this i.c. inoculation route, we previously found that by 8 dpi the virus has spread throughout the brain parenchyma, although its level of replication is highest in the ependyma [30, 31]. Following clustering, with the each cluster represented by a number, we were able to identify clusters representing oligodendrocytes (via double Mobp and Mbp positivity, cluster 23), OPCs (via double Pcdh15 and Pdgfra positivity, clusters 4,25,30,32,33,35), myeloid cells (via double Csf1r and Cx3cr1 positivity, clusters 3,17,27,42,34), Cd8 T cells (via double Cd8a and Cd8b1 positivity, cluster 24), and ependymal cells (via double Cfap65 and Foxj1 positivity, cluster 28) (Fig. 1B). Furthermore, we were able to observe that the ependyma specifically, cluster 28, in the infected condition appeared to be uniquely enriched in LTag and VP1 viral transcripts compared to every other cell type, demonstrating active viral infection (Fig. 1B).

Concerns with contamination from transcripts of colocalized CD8^+^ T and/or myeloid cells led us to carry out additional filtering on the spots categorized as ependymal by selecting for Foxj1/Cfap65-high and Cd8a/Cd8b1/Csf1r/Cx3cr1-low cells. Furthermore, spatial data showed that there was a clear presence of choroid plexus epithelial-localized transcripts in the “ependyma” cluster, so choroidal spots were removed via spatial selection. Finally, to further address concerns of contamination stemming from co-localized immune cells, genes present in either over 35% of spots classified as CD8^+^ T or over 35% of spots classified as myeloid were removed from consideration (a total of 18 genes were removed in this fashion). Following this filtering, DEG analysis was conducted, revealing a major change in the gene profile biasing towards upregulation with 36 significantly upregulated genes and 16 significantly downregulated (of 122 total) (Fig. 1C). Although Cxcl12 and Cxcl16 transcripts were not among the upregulated genes, expression of both is visible along the ependyma in both sham and infected mice, indicating constitutive basal expression of these chemokines (Fig. 1D). Due to previous findings demonstrating the impact of CXCR4 and CXCR6 on recruitment of T cells to the brains of mice infected with West Nile Virus (WNV), the expression of these specific chemokine ligands are shown [32, 33].

Consistent with our prior studies, transcripts for CD8^+^ T cells were noticeably present in brain sections from MuPyV-infected mice [31]. Again, due to concerns with contamination from transcripts of colocalized ependymal or myeloid cells, further filtering was done on the spots categorized as CD8 T cells, by selecting for Cd8a/Cd8b1-high and Foxj1/Cfap65/Csf1r/Cx3cr1-low cells. Following this filtering, levels of expression of various genes relevant to CD8^+^ T cell function were assessed in the infected condition, revealing upregulation of Gzmb, Cxcr3, Cxcr6, Ccr2, Ifnar1, and Ifngr1 (Fig. 1E and F). Cxcr4 was seen at low levels along the ventricles, while highly upregulated Cxcr6 expression was seen in the same area as well as the meninges (Fig. 1F). Finally, to investigate other possible pathways for ligand/receptor interaction between ependymal and CD8^+^ T cells, we queried a previously established database of ligand-receptor interactions in mice [34]. This revealed possible interactions between Cxcr6 in T cells and various chemokines found to be upregulated by ependymal cells following inflammation, such as CXCL10, CCL5, CXCL9, and CX3CL1 (Fig. 1G).

To confirm the location of infection, wild-type (WT) mice were i.c. injected with MuPyV or vehicle, and at 7 dpi the periventricular (PVR) and non-periventricular regions (non-PVR) of the brain were dissected (Fig. 1H). Transcripts specific to the ependyma, *Foxj1* and *FAM183b*, were measured via qPCR in the sham mice, confirming expression only in the PVR (Fig. 1I). Nearly 2-log higher expression of LTag mRNA was detected in the PVR than non-PVR (Fig. 1J). Taken together, transcriptomic analyses confirm that MuPyV CNS infection primarily involves the ependyma which assumes a marked inflammatory state, including expression of several chemokine genes that may interact with chemokine receptors expressed by co-localized CD8^+^ T cells.

**Fig. 2:**
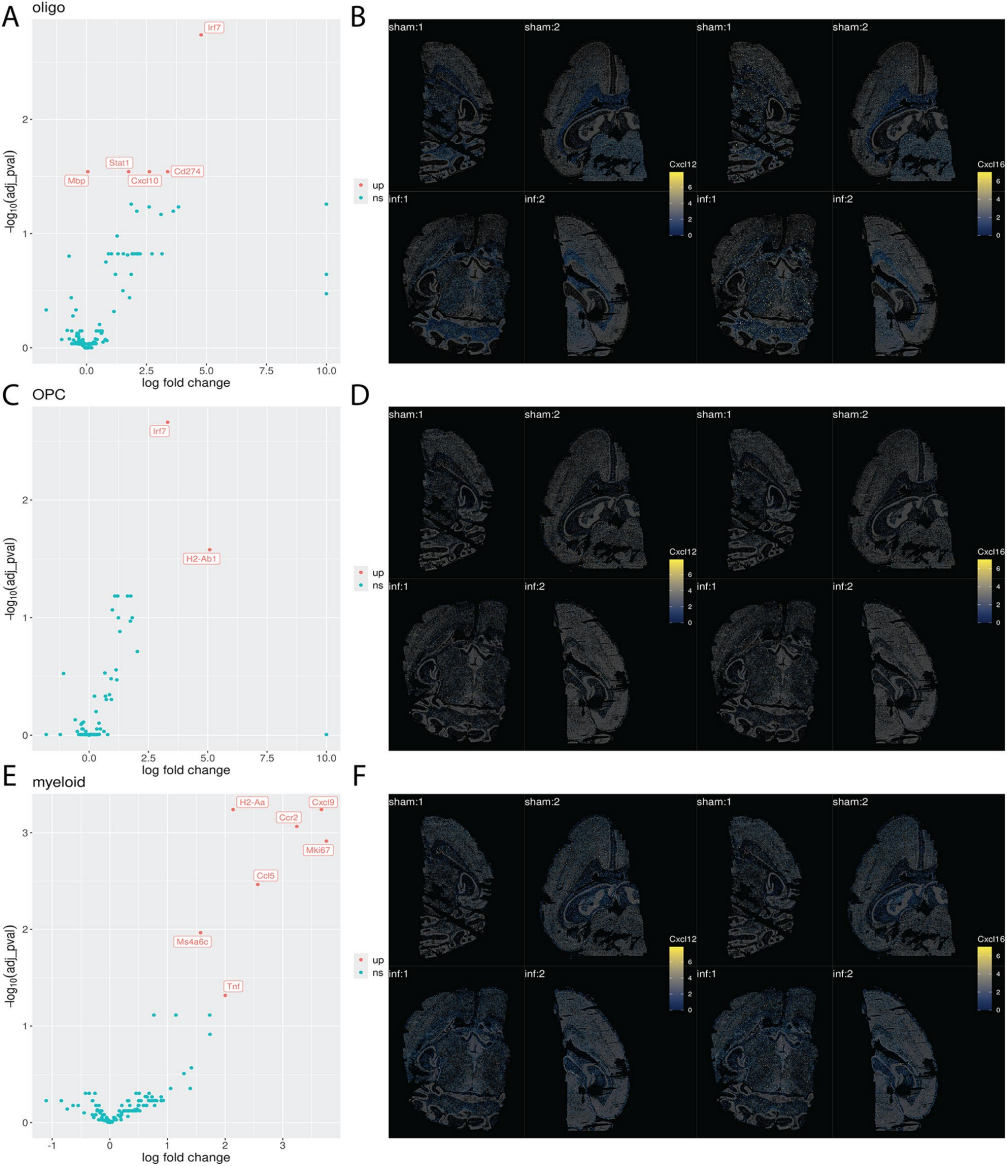
Spatial transcriptomics of oligodendrocytes, OPCs, and myeloid cells. **A**. Volcano plot displaying DEG analysis for oligodendrocytes. Each gene is represented by a labeled point, organized along the x-axis according to computed log-fold change and by the negative log of the adjusted *p* value on the y-axis. Points are colored according to the nature of the dysregulation (up, down, or not significant). **B**. Expression of Cxcl12 and Cxcl16 transcripts in oligodendrocytes in sham and infected samples. Spots are placed according to their x and y coordinates, ignoring their z coordinate (i.e., flattening). Oligodendrocyte spots are colored according to log-normalized Cxcl12 and Cxcl16 transcript expression, with non-oligodendrocyte spots greyed out and made semi-transparent. **C**. Volcano plot displaying DEG analysis for OPCs. Each gene is represented by a labeled point, organized along the x-axis according to computed log-fold change and by the negative log of the adjusted *p* value on the y-axis. Points are colored according to the nature of the dysregulation (up, down, or not significant). **D**. Expression of Cxcl12 and Cxcl16 transcripts in OPCs in sham and infected samples. Spots are placed according to their x and y coordinates, ignoring their z coordinate (i.e., flattening). OPC spots are colored according to log-normalized Cxcl12 and Cxcl16 transcript expression, with non-OPC spots greyed out and made semi-transparent. **E**. Volcano plot displaying DEG analysis for myeloid cells. Each gene is represented by a labeled point, organized along the x-axis according to computed log-fold change and by the negative log of the adjusted *p* value on the y-axis. Points are colored according to the nature of the dysregulation (up, down, or not significant). **F**. Expression of Cxcl12 and Cxcl16 transcripts in myeloid cells in sham and infected samples. Spots are placed according to their x and y coordinates, ignoring their z coordinate (i.e., flattening). Myeloid spots are colored according to log-normalized Cxcl12 and Cxcl16 transcript expression, with non-myeloid spots greyed out and made semi-transparent

Oligodendrocytes, OPCs, and myeloid identity assigned clusters were also analyzed. The myeloid group was further filtered for Csf1r/Cx3cr1-high and Foxj1/Cfap65/Csf1r/Cx3cr1-low cells to address concerns surrounding ependymal or CD8^+^ T cell transcript contamination. A similar gene filtering process as described previously in the ependymal differentially expressed gene (DEG) analysis was conducted to remove CD8^+^ T or ependymal contamination in the myeloid cell DEG analysis. While oligodendrocytes and OPCs did not show a major change in their gene profile between sham and infected mice, besides a modest upregulation of antigen presentation and interferon-associated genes, a pro-inflammatory response was observed in myeloid cells (Fig. 2A, C and E). No significant differential expression of Cxcl12 or Cxcl16 transcripts between sham and infected conditions, however, was appreciated in any of these cell populations, although a basal level of expression was discerned in each these cell lineages (Fig. 2A, B, C, D, E and F).

### Expression of CXCR4 and CXCR6 on T cells is specific to the MuPyV- infected brain

**Fig. 3:**
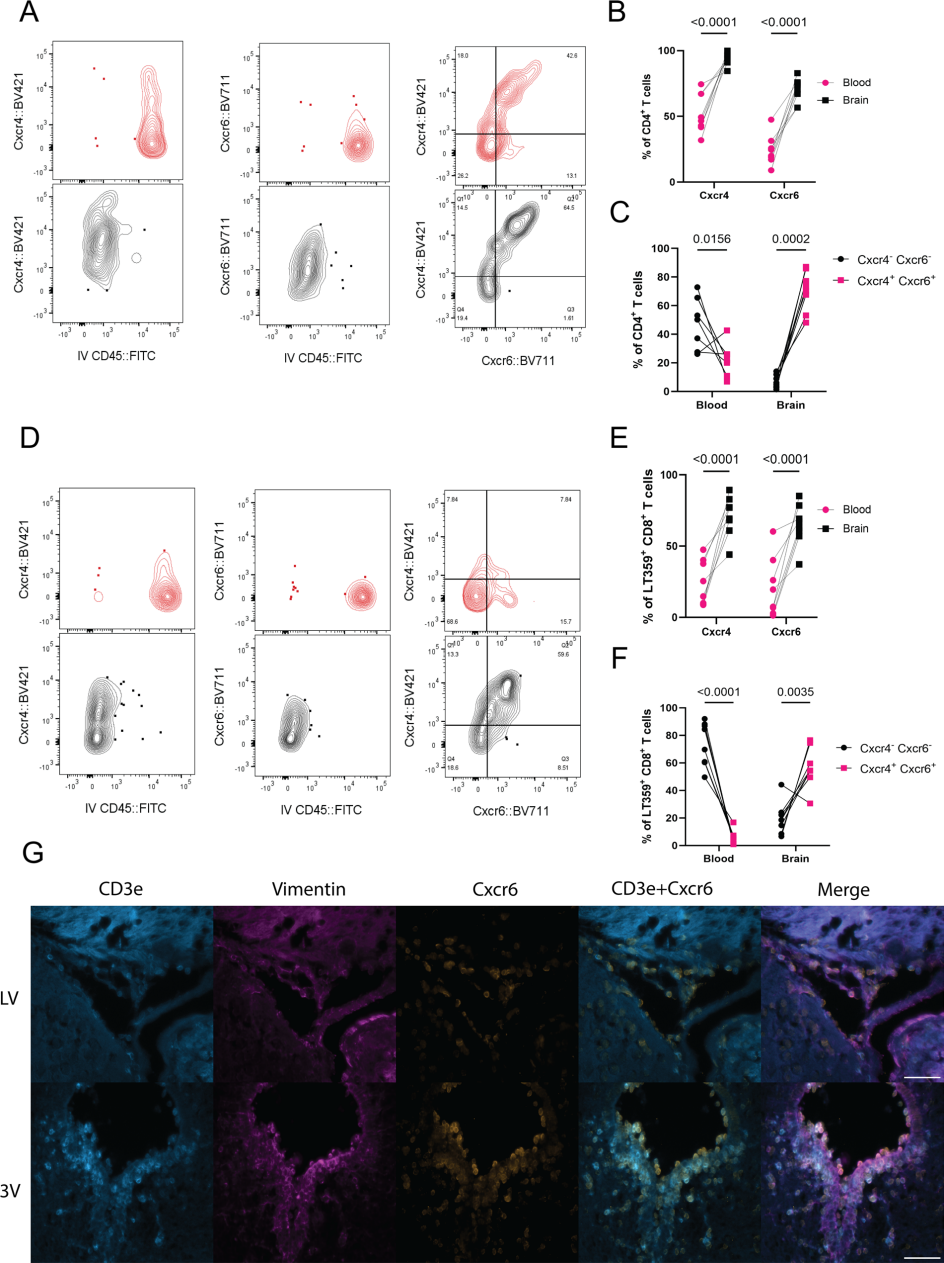
Expression of CXCR4 and CXCR6 on T cells after MuPyV infection is brain-specific. WT mice were inoculated i.c. with MuPyV and IV labeled with CD45::FITC prior to euthanasia. Flow cytometry of the indicated C-C and C-X-C receptors on brain CD4^+^ and D^b^ LT359 tetramer^+^ CD8a^+^ T cells. **A**. Representative contour plots showing brain CD4^+^ T cells have increased CXCR4 and CXCR6 staining compared to IV CD45^+^ cells in the blood. More brain than blood cells were CXCR4^+^ CXCR6^+^. **B-C.** Quantification of the percent positive cells in **A. D**. As with CD4^+^ T cells, brain D^b^ LT359 tetramer^+^ CD8^+^ T cells have increased CXCR4 and CXCR6 staining, as well as CXCR4^+^ CXCR6^+^ cells. **E-F**. Quantification of the percent positive cells in **D. G**. Representative images of 8 dpi WT mouse brains at the third ventricle (3 V) and lateral ventricle (LV) stained for CD3e, vimentin, and CXCR6. Both CXCR6^+^ and CXCR6^+^ CD3e^+^ cells are visible at the ventricles. Flow cytometry of *n* = 8 mice for two independent experiments. Scale bar 50 μm. Percent positive values were determined using the fluorescence minus one (FMO) values for each chemokine. The *p*-values shown are matched 2-way ANOVA with post-hoc

To confirm the expression of chemokine receptors on T cells identified by MERFISH spatial transcriptomics, flow cytometry was performed using WT mouse brains and blood samples taken at 8 dpi, with IV injection of FITC-conjugated anti-CD45 to distinguish vascular from parenchymal cells [35](Supp. Figure 1). The receptors analyzed represent chemokine receptors involved in T cell trafficking to the brain: CCR4 [36, 37], CCR5 [38–40], CCR6 [41, 42], CCR7 [38, 43], CCR9 [44, 45], CXCR3 [46, 47], CXCR4 [33, 48], CXCR5 [49], and CXCR6 [50–53]. The expression of several chemokine receptors was elevated on the brain-resident CD4^+^ and virus-specific (i.e., D^b^ LT359 tetramer^+^) CD8^+^ T cells relative to the blood, including CCR5, CCR6, CCR7, CXCR3, CXCR4, and CXCR6 for CD4^+^ T cells (Fig. 3A-B, Supp. Figure 1B-D) and CCR6, CCR7, CXCR4, and CXCR6 on virus-specific CD8^+^ T cells (Fig. 3D-E, Supp. Figure 1E-G). Interestingly, the majority of the brain CD4^+^ and CD8^+^ T cells co-expressed CXCR4 and CXCR6, whereas the blood CD4^+^ and CD8^+^ T cells were CXCR4^−^ CXCR6^−^ (Fig. 3C, F). Instead, the brain appears to recruit and maintain T cells via several chemokine receptors. The spatial localization of these T cells was confirmed by immunofluorescence staining of brain sections for CXCR6 and CD3e, along with anti-vimentin to label the ependyma (Fig. 3G). The majority of CD3e^+^ cells overall and those expressing CXCR6 were located in the PVR.

### Loss of CXCR6 or CXCR4 individually is insufficient to alter T cell migration to the brain after MuPyV infection

**Fig. 4:**
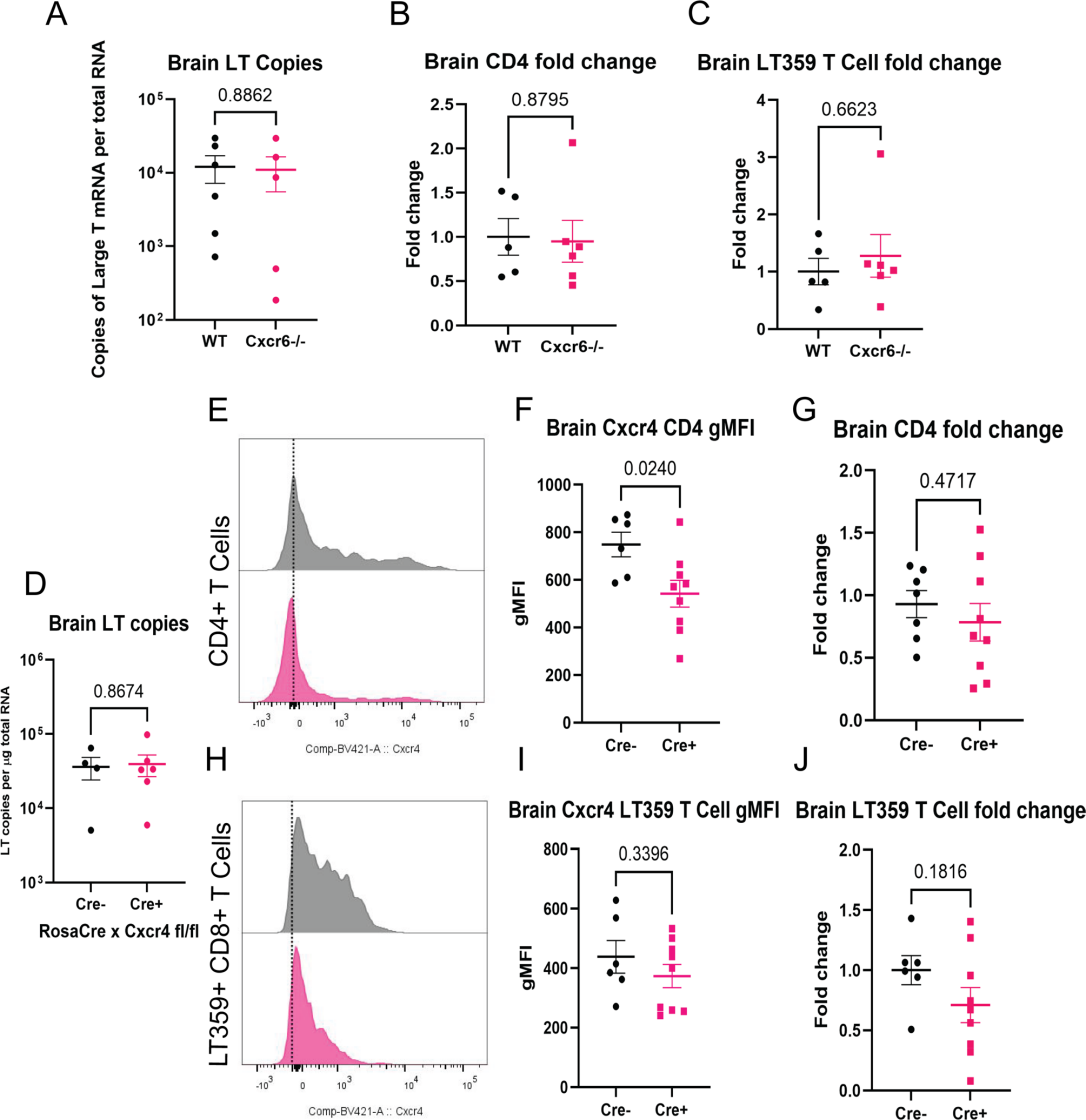
Loss of CXCR6 or CXCR4 signaling alone has no effect on recruitment of T cells to the brain. **A-D**. WT or CXCR6^−/−^ mice were inoculated i.c. with MuPyV and euthanized at 8 dpi. Tissues were used for flow cytometric analysis of T cell numbers and expression of CXCR4, as well as qPCR to determine virus levels. *n* = 6 (WT) or 7 (CXCR6^−/−^) mice per group, 2 independent experiments. (**A)** LT mRNA copies by qPCR of WT or CXCR6^−/−^ mice. Number of CD4^+^ T cells (**B**) or D^b^ LT359 tetramer^+^ CD8^+^ T cells (**C**) in the brains of WT and CXCR6^−/−^ mice calculated as fold change relative to the mean of the WT controls per experiment. **D-J.** Rosa-Cre^ERT^ x Cxcr4^fl/fl^ (Cre^+^ and Cre^−^) mice were treated with tamoxifen for 5 d, then inoculated with MuPyV i.c. and euthanized at 8 dpi. Tissues were used for flow cytometry and qPCR. **D.** LT mRNA copies of Cre^−^ and Cre^+^ Rosa-Cre^ERT^ x Cxcr4^fl/fl^ brains. **E**,** H.** Representative histograms of Cre- and Cre + expression of CXCR4 on CD4^+^ T cells (**E**) or D^b^ LT359 tetramer^+^ CD8^+^ T cells (**H**). **F**,** I.** gMFI of CXCR4 expression by CD4^+^ T cells (**F**) or CD8^+^ T cells (**I**). **G**,** J.** Fold change of the number of CD4^+^ T cells (**G**) or D^b^ LT359 tetramer^+^ CD8^+^ T cells (**J**) were calculated relative to the Cre^−^ controls, *n* = 6 (Cre-) or 10 (Cre^+^) mice per group. Data analyzed by two-tailed Student’s *t* test

To assess the effect of loss of CXCR6 on the T cell response to MuPyV CNS infection, WT and Cxcr6^−/−^ mice were infected i.c. with MuPyV, and T cell numbers and LTag mRNA levels were analyzed at 8 dpi. Absence of CXCR6 did not affect the level of infection (Fig. 4A) or CD4^+^ or virus-specific CD8^+^ T cell numbers (Fig. 4B-C) in the brain. Similarly, no changes in LTAg transcript levels or T cell numbers were seen in the spleen and cervical lymph nodes. (Supp. Figure 2A-C). Thus, CXCR6 loss alone did not abrogate T cell entry and maintenance during MuPyV infection.

As the CD4^+^ and virus-specific CD8^+^ T cells in the brain also expressed CXCR4, we asked whether CXCR4 was responsible for T cell migration and maintenance. Because *Cxcr4*^−/−^ is embryonically lethal, we utilized a ROSA-Cre^ERT^ x Cxcr4^fl/fl^ mouse system to induce deletion of *Cxcr4* prior to infection by administering tamoxifen; brain, cervical lymph nodes, and spleen were harvested 8 dpi. The expression of CXCR4 on CD4^+^ and virus-specific CD8^+^ T cells was reduced in the spleen, cervical lymph node, and brain (Supp. Figure 2E-F, H-I, L-M, O-P, Fig. 4E-F, H-I). The reduction in CXCR4 expression had no effect on CD4^+^ or virus-specific CD8^+^ T cells in any tissue (Fig. 4G, J, Supp. Figure 2G, J, N, Q) nor LT copies (Fig. 4D, Supp. Figure 2D, K). As with loss of CXCR6, loss of CXCR4 alone was insufficient to disrupt T cell migration to the brain following MuPyV infection.

### Combined loss of CXCR6 with inhibition of CXCR4 increased MuPyV-induced T cell recruitment

**Fig. 5:**
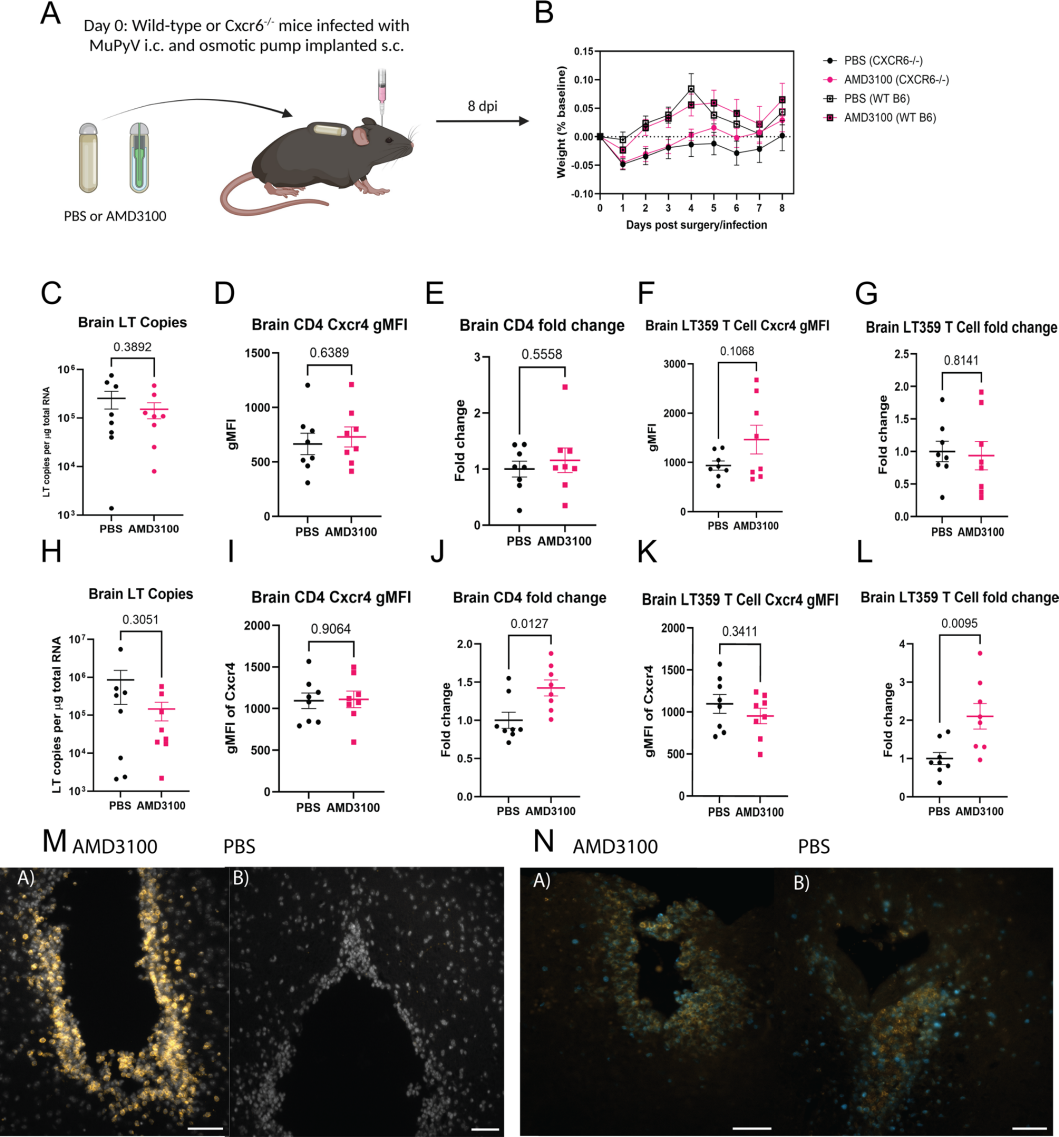
Inhibition of CXCR4 combined with loss of CXCR6 increases brain T cell recruitment. **(A)** Experimental paradigm. WT or CXCR6^−/−^ mice were inoculated i.c. with MuPyV and simultaneously implanted with osmotic pumps filled with either PBS or AMD3100 Mice were weighed daily, then euthanized at 8 dpi, and tissues were collected for flow cytometry and qPCR. Diagram made using BioRender.com. **(B)** Weights of the mice as percent of baseline during the experiments. **C**,** H.** LT copies by RT qPCR in WT (**C**) and Cxcr6-/- (**H**) mice treated with PBS or AMD3100. **D**,** I.** gMFI of Cxcr4 in CD4 + T cells in WT (**D**) or Cxcr6-null (**I**) mice with drug treatment. **E**,** J.** Fold change of CD4 + T cells relative to PBS controls in WT (**E**) and CXCR6-/- (**J**) mice. **F**,** K.** gMFI of CXCR4 in D^b^ LT359 tetramer^+^ CD8^+^ T cells in WT (**F**) or CXCR6-/- (**K**) mice. **G**,** L.** Fold change of virus-specific CD8^+^ T cells relative to PBS controls in WT (**G**) and CXCR6^−/−^ (**L**) mice. **M**. Increased CD8^+^ T cells (orange) at the ependyma in CXCR6^−/−^ mice with AMD3100 treatment (a) compared to PBS controls (b). **N.** In CXCR6^−/−^ mice, CD8^+^ T cells (orange) show Ki67 (blue) positivity after both AMD3100 treatment (a) and PBS treatment (b). *n* = 3 mice per group, two independent experiments. Scale bar 50 μm. Data analyzed by two-tailed Student’s *t* test

Because CXCR4 and CXCR6 were largely co-expressed on brain CD4^+^ and virus-specific CD8^+^ T cells of MuPyV-infected mice, we considered the possibility that both receptors could regulate T cell recruitment. The antagonist AMD3100 (plerixafor) inhibits CXCL12 activity at the receptor without inhibiting related C-X-C receptors [54]. Alzet osmotic pumps were loaded with either PBS or AMD3100 to deliver at a rate of 10 mg/kg/day for 8 days. Both WT and Cxcr6^−/−^ mice were i.c. injected with MuPyV, and the osmotic pumps were then implanted subcutaneously (Fig. 5A). Mice were weighed daily to ensure recovery (Fig. 5B). At 8 dpi, tissues were collected for flow cytometry and qPCR analyses. There was no change to CXCR4 GMFI due to this being a blockade model and not a genetic knockout as shown earlier (Fig. 5. D, F, I, K). As in the ROSA-Cre^ERT^^+^ x Cxcr4^fl/fl^ mice, the WT mice receiving AMD3100 show no changes in virus levels based on copies of LTag mRNA compared to PBS controls (Fig. 5C, Supp. Figure 3B, G). Furthermore, there were no differences in CD4^+^ T cells or virus-specific CD8^+^ T cells in any tissue (Fig. 5E, G, Supp. Figure 3E, F, I, K), further demonstrating that loss or inhibition of CXCR4 alone is inadequate to affect T cell migration.

In contrast, Cxcr6^−/−^ mice implanted with AMD3100 pumps showed a significant increase in numbers of brain CD4^+^ T cells and virus-specific CD8^+^ T cells compared to CXCR6^−/−^ PBS controls (Fig. 5J and L). No increase in T cell numbers was seen in the spleen or the lymph nodes of the same mice (Supp. Figure 3P and 3U), demonstrating this effect is specific to the brain. The copies of LTag mRNA were also not significantly different (Fig. 5H).

This increase in CD8^+^ T cells was also visualized by IF microscopy. Whereas the PBS-treated mice had some CD8^+^ T cells in the choroid plexus and at the third ventricles, a higher proportion of these cells were seen at the ependyma and choroid plexus, third ventricles, and lateral ventricles in the AMD3100-treated, Cxcr6^−/−^ mice (Fig. 5M). In both treatment groups, CD8^+^ T cells found along ventricles were Ki67^+^, indicating a proliferative state (Fig. 5N). The results of this experiment point to a functional non-redundancy between the signaling axes of CXCR6/CXCL16 and CXCR4/CXCL12 to limit brain T cell recruitment and migration to MuPyV CNS infection.

## Discussion

Entry of virus-specific T cells into the brain must be finely tuned to control infection while mitigating injury in an organ replete with essential, nonrenewable cells. Using the MuPyV CNS infection model to explore early events in the pathogenesis of JCPyV-PML, we determined that brain-infiltrating CD4^+^ and CD8^+^ T cells co-express the CXCR4 and CXCR6 chemokine receptors, which act in concert to limit T cell aggregation at the infected ependyma. Spatial transcriptomics has recently been applied to profile changes in the brains of adult and aged mice to West Nile Virus infection [55]. Here, spatial transcriptomics confirmed and extended our previous immunofluorescence microscopy analyses showing that the ependyma is the major site of productive MuPyV replication during acute infection of the mouse brain.

MERFISH profiled innate immune responses by the ependyma to MuPyV infection, including pronounced upregulation of transcripts for STAT1, STAT2, and CXCL10. In this connection, CNS infection of STAT1 knockout mice with MuPyV results in dramatic disruption of the ependyma and high virus levels [31]. CXCL12 and CXCL16 are expressed by the ependyma and periventricular region irrespective of infection, paralleling the expression of CXCR4 and CXCR6 by brain-infiltrating T cells to regulate their migration to infectious foci to the ventricular barrier. The CXCR3-CXCL9/10 chemokine axis is commonly used to direct T cells to inflamed non-lymphoid tissues. Although MERFISH analyses showed upregulation of CXCR3 mRNA in total CD8^+^ T cells and CXCL9 and CXCL10 transcripts by MuPyV-infected ependymal cells, virus-specific CD8^+^ T cells did not express CXCR3. Non-MuPyV CXCR3^+^ CD8^+^ T cells may be recruited to the periventricular region consequent to anti-MuPyV CD8^+^ T cells engaging infected ependyma and secreting IFN-g to induce ependymal production of CXCL9 and CXCL10. In line with this possibility, effector-phenotype transcripts (e.g., granzyme b and IFN-g) are detected in CD8^+^ T cells localized at the ependyma. Notably, the infected ependyma also downregulate Cfap65 mRNA encoding cilia and flagella associated protein 65 and Cldn5 mRNA for the tight junction protein Claudin-5, suggesting that MuPyV infection and/or periventricular anti-viral T cells negatively impact the integrity of the ependymal barrier.

Chemokine-chemokine receptor axes have been shown to be instrumental in regulating cell-mediated control of several CNS viral infections. In murine infection models with Japanese encephalitis virus, recruitment of T-cells is mediated via CCR5 and CCR2 [39, 56]. CXCR4 and CXCR6 have been implicated in this role during WNV infection of the CNS. CXCL12 produced by the endothelium during WNV infected to retain T cells in the perivascular space, a loss of CXCR4 allows T cells to breach the BBB [32, 33]. CXCR4 and CXCR6 individually regulate the migration of T cells in this infection model. In the case of MuPyV, both CXCR4 and CXCR6 inhibit entry into the brain parenchyma; however, the MEFISH data did not detect viral transcripts in brain endothelium (Supp. Fig. 4A) or significant expression of CXCL12 and CXCL16 in non-ependymal cells. CD4^+^ and CD8^+^ T cells have been shown to enter the brain through the choroid plexus in both inflammatory and homeostatic conditions [42, 57]. An intriguing possibility is that CXCR4 and CXCR6 act as gatekeepers to limit MuPyV-specific T cells entry across the BCSF barrier to reach the CXCL12- and CXCL16-producing ependyma.

A combined requirement for two chemokine receptors to regulate virus-specific T cell CNS entry may be needed to control MuPyV infection of the ependyma while limiting damage to this critical brain barrier. Periventricular MuPyV-specific T cells are highly proliferative, as indicated by Ki67 positivity, regardless of the blockade of CXCR4 or loss of CXCR6. With dual CXCR4 and CXCR6 expression being specific to brain-infiltrating T cells, we speculate that the increased of CD4^+^ and CD8^+^ T cell numbers after loss of signaling by these specific chemokine receptors is a brain-localized effect. Evidence from other groups suggests that these receptors have different effects on T cell differentiation and functionality. Loss of CXCR4 reverses the exhausted T cell phenotype in vivo and is often downregulated upon T cell activation [58–60]. Loss of CXCR6 has been reported to promote clonal expansion of CD8^+^ T cells in the brain [61]. These findings suggest that CXCR4 and CXCR6 act to restrain T cell proliferation and effector functions, which would be critical in checking immunopathologic damage in the CNS. Because T cell entry into the CNS in other viral systems has been shown to be affected by loss of individual chemokine receptors, our findings argue that the type of viral infection, and the location and cell sources of chemokines, dictate the profile of chemokine receptors expressed by brain-infiltrating T cells.

Even with the same microbial pathogen, organ-specific differences come into play in shaping the chemokine receptors engaged to modulate T cell infiltration. The involvement of two chemokine signaling axes speaks to their functional non-redundancy, and to their involvement in limiting T cell entry into the MuPyV-infected CNS. In contrast, loss of CXCR6 by kidney-infiltrating T cells or neutralization of CXCL16 during MuPyV infection are sufficient to impede entry into this organ [53]. Indeed, differences in chemokine receptor expression profile of brain and kidney T cells responding to the same pathogen points to their tissue-specific recruitment and regulation. Similar context- and subset-specificity has been recently described for myeloid cells, where the function of chemokine receptors is redundant in the establishment of tissue-residency, but is not redundant for recruitment to sites of inflammation [62–64]. Understanding the interplay by T cell chemokine receptors may identify subsets of cells important for tissue-specific immune responses.

Anti-CXCR6 antibody therapy has been proposed for the treatment of MS via deletion of CD4^+^ T cells, as have anti-CXCL16 therapeutics [65]. Currently, CXCR4 antagonists are approved for cancer treatment, including revamping the antitumor response after immune modulation by hepatocellular carcinoma [66]. Pancreatic and breast cancer models have also shown improvement after CXCR4 blockade [67]. Beyond cancer, a study of acute myocardial infarction in mice discovered an elevated T cell-mediated recovery rate after treatment with the CXCR4 antagonist POL5551 [68]. The combination of CXCR6 and CXCR4 antagonism shown in our studies suggests a potential way for improving PML immunotherapy, instead of use in blockade therapy [69, 70]. As cells nonproductively and productively infected with JCPyV express the viral LT antigen, and a higher proportion of MuPyV-infected ependymal cells express LT transcripts than capsid VP1 transcripts. LT-specific T cells would recognize infected cells regarding of the state of infection. Adoptive JCPyV-specific T cell therapy for PML is already in clinical trials [70]. Our findings suggest that handicapping signaling by select chemokine receptors expressed by donor JCPyV-specific T cells may facilitate their migration to infected glia [71].

The double-edged sword of successful control of infection by antiviral T cells is the potential for injurious neuroinflammation [72, 73]. IFN-g from activated CD8^+^ T cells has been reported to damage OPCs [74]. T cells also activate microglia, leading to the pruning of synapses in WNV and Zika virus infections [75, 76]. Excess secretion of chemokines, such as CCL5 during mouse hepatitis virus CNS infection or CXCL1 in MS, can directly contribute to demyelination [77, 78]. Understanding pathways that limit T cell entry into the CNS is important in minimizing injury to neural tissues; for early JCPyV infection, this goal would be particularly critical in preserving the SVZ neurogenic niche. From a broader perspective, T cell-driven neuroinflammation, possibly in response to viral infection, may be involved in the initiation or progression of neurodegenerative disease [79–81].

In summary, this study establishes the ependyma as a central site of MuPyV infection and inflammation-associated transcriptomic changes. Immune responses in the CNS during any viral infection must negotiate migration and activation of virus-specific T cells against deleterious inflammation, including that caused by innate and adaptive antiviral countermeasures. Although a number of individual chemokine receptors have been studied for their contribution to T cell regulation, the interplay between multiple chemokines remains a major gap in neuroimmunology. Our work demonstrates the importance of the dual regulation by CXCR4 and CXCR6 on T cell movement in a MuPyV infection system. Our work defining chemokine receptor-chemokine axes required for virus-specific T cell control should inform future modifications of adoptive T cell therapies for CNS viral infections.

### Study limitations

There are a number of limitations to our study. The MERFISH analyses were performed at only a single acute postinfection timepoint (8 dpi). It would be instructive to also compare gene expression profiles at infection timepoints prior to infiltration of virus-specific T cells (e.g., 4 dpi), as well as during persistent MuPyV infection. Spatial transcriptomics used here involved a customized 140 gene expression panel focusing on detecting transcripts for chemokines, chemokine receptors, and innate immune genes; a more extensive gene panel could reveal additional differences in the transcriptional landscape of the brain in response to MuPyV infection. Our study also did not assess potential recruitment of non-T cell populations expressing CXCR4 and CXCR6 during MuPyV encephalitis. Finally, the impact of excessive T cell infiltration in the intrinsic co-absence of CXCR4 and CXCR6 on neuropathology, as well as defining T cell effector mechanisms causing neural injury, during MuPyV CNS infection merit exploration.

## Electronic supplementary material

Below is the link to the electronic supplementary material.

**Supplementary Material 1**: **Supplemental Fig. 1**. Brain-specific expression of chemokine receptors on CD4 and CD8 T cells following mupyv infection. WT mice were inoculated i.c. with MuPyV and IV labeled with CD45::FITC prior to euthanasia. Flow cytometry of CCR4, CCR5, CCR6, CCR9, CXCR3 and CXCR5 were assessed. (A) Flow gating strategy. FVD = fixable viability dye. Gating for Cxcr4 + and Cxcr6 + cells were based on fluorescence minus one (FMO) values, and are representative of the gating strategies used for all experiments. FMOs are represented by the dashed line. (B) Representative contour plots depicting expression of CCR5, CCR6, CCR7, and CXCR3 in brain CD4^+^ T cells. C-D. Quantification of B for C-C receptors (C) and C-X-C receptors (D). E. Expression of CCR6 and CCR7 was increased in brain D^b^ LT359^+^ CD8^+^ T cells. F-G. Quantification of E for C-C receptors (F) and C-X-C receptors (G). Flow cytometry, *n* = 8 mice. Percent positive values were determined using the FMO values for each chemokine. The *p*-values shown are matched 2-way ANOVA with post-hoc analysis.

**Supplementary Material 2: Supplemental Fig. 2.** Loss of either CXCR6 or CXCR4 does not affect Splenic or cervical lymph node T cell numbers after mupyv infection. A-C. WT or CXCR6−/− mice were inoculated i.c. with MuPyV and euthanized at 8 dpi. Tissues were used for flow cytometric analysis of T cell numbers and expression of CXCR4, and qPCR to determine virus levels. *n* = 6 (WT) or 7 (CXCR6−/−) mice per group, 2 independent experiments. A. LT mRNA copies by qPCR in the spleen and cervical lymph nodes of WT or CXCR6−/− mice. B-C. Number of CD4+ T cells (B) or Db LT359+ CD8+ T cells (C) in the spleens of WT and CXCR6−/− mice calculated as fold change relative to the mean of the WT controls per experiment. D-Q. Rosa-Cre^ERT^ x Cxcr4fl/fl (Cre+ and Cre−) mice were treated with tamoxifen for 5 d, then i.c. inoculated with MuPyV and euthanized at 8 dpi. Tissues were used for flow cytometry and qPCR. D, K. LT mRNA copies by qPCR in the spleen (D) and cervical lymph nodes (K) of Cre− or Cre+ Rosa-Cre^ERT^ x Cxcr4fl/fl mice. E, H. Representative histograms of Cre− and Cre+ expression of CXCR4 on CD4+ T cells (E) or Db LT359 tetramer+ CD8+ T cells (H) in the spleen. F, I. gMFI of splenic CXCR4 for CD4+ T cells (F) or Db LT359 tetramer+ CD8+ T cells (I) by Cre expression. G, J. Fold change of the number of CD4+ T cells (G) or Db LT359 tetramer+ CD8+ T cells (J) in the spleen calculated relative to the Cre− controls. L, O. Representative histograms of Cre− and Cre+ expression of CXCR4 on CD4+ T cells (E) or Db LT359 tetramer+ CD8+ T cells (H) in the cervical lymph node. F, I. gMFI of lymph node CXCR4 for CD4+ T cells (F) or Db LT359 tetramer+ CD8+ T cells (I) by Cre expression. G, J. Fold change of the number of CD4+ T cells (G) or Db LT359 tetramer+ CD8+ T cells (J) in the cervical lymph nodes calculated relative to the Cre- controls. n = 6 (Cre−) or 10 (Cre+) mice per group. Data analyzed by a two-tailed Student’s t test.

**Supplementary Material 3**: **Supplemental Fig. 3**. Inhibition of CXCR4 combined with loss of CXCR6 does not impact spleen or cervical lymph node T cell numbers. WT or CXCR6^−/−^ mice were inoculated i.c. with MuPyV and simultaneously implanted with osmotic pumps filled with either PBS or AMD3100. Mice were weighed daily then euthanized at 8 dpi and tissues were collected for flow cytometry and qPCR. A. Flow cytometry gating strategy. B, G. LT mRNA copies by qPCR in the spleens (B) or cervical lymph nodes (G) of WT mice treated with AMD3100 or PBS. C, H. gMFI of CXCR4 on CD4^+^ T cells in WT mouse spleens (C) or lymph nodes (H). D, I. Fold change of CD4^+^ T cells relative to PBS controls in spleens (D) and lymph nodes (I) from WT mice. E, J. gMFI of CXCR4 in virus-specific CD8 + T cells in WT spleens (E) or lymph nodes (J). F, K. Fold change of D^b^ LT359 tetramer^+^ CD8^+^ T cells relative to PBS controls in spleens (F) and lymph nodes (K) of WT mice. L, Q. LT mRNA copies by qPCR in the spleens (L) or cervical lymph nodes (Q) of CXCR6^−/−^ mice treated with AMD3100 or PBS. M, R. gMFI of CXCR4 on CD4^+^ T cells in CXCR6^−/−^ spleens (M) or lymph nodes (R). N, S. Fold change of CD4^+^ T cells relative to PBS controls in spleens (N) and lymph nodes (S) from CXCR6^−/−^ mice. O, T. gMFI of CXCR4 in D^b^ LT359 tetramer^+^ CD8^+^ T cells in CXCR6^−/−^ spleens (O) or lymph nodes (T). P, U. Fold change of D^b^ LT359 tetramer^+^ CD8^+^ T cells relative to PBS controls in spleens (P) and lymph nodes (U) of CXCR6^−/−^ mice. *n* = 8 mice per group, 2 independent experiments. Data analyzed with a two-tailed Student’s t test.

**Supplementary Material 4**: **Supplemental Fig. 4**. Spatial transcriptomic analysis shows that CD8^+^ T cells do not localize with endothelium in brains of MuPyV-infected mice. A. MERFISH transcript visualization shows CD8a (blue), CD4 (green), Cldn5 (red), Flt1 (yellow), and LT antigen (white).

**Supplementary Material 5**

## Data Availability

All code relevant to this publication has been made available here: https://github.com/stratton-lab/alexander-2025-cxcr. Raw MERSCOPE outputs have been uploaded to the GEO omnibus, GEO accession code: https://www.ncbi.nlm.nih.gov/geo/query/acc.cgi?&acc=GSE296357. Private reviewer token: ixknemgwhxedzqj.

## Abbreviations

IC: Intracranial
IV: Intravenous
CNS: Central nervous system
MuPyV: Mouse polyomavirus
PML: Progressive Multifocal Leukoencephalopathy
MERFISH: Multiplexed error-robust fluorescence in situ hybridization
BBB: Blood-brain barrier
BCSF: Blood-cerebrospinal fluid (CSF)-ependyma barrier
CSF: Cerebrospinal fluid
EVs: Extracellular vesicles
PVR: Periventricular region
IF: Immunofluorescence
LT: Large T antigen
LV: Lateral Ventricle
3 V: Third Ventricle
SVZ: Subventricular zone
OPCs: Oligodendrocyte precursor cells
