# Supplementary figures and images for: CXCR4 and CXCR6 dually limit T cell entry into the polyomavirus-infected brain
Source: J Neuroinflammation. 2025 Jun 28;22:169. doi: 10.1186/s12974-025-03496-2 (PMC12205503; doi:10.1186/s12974-025-03496-2)

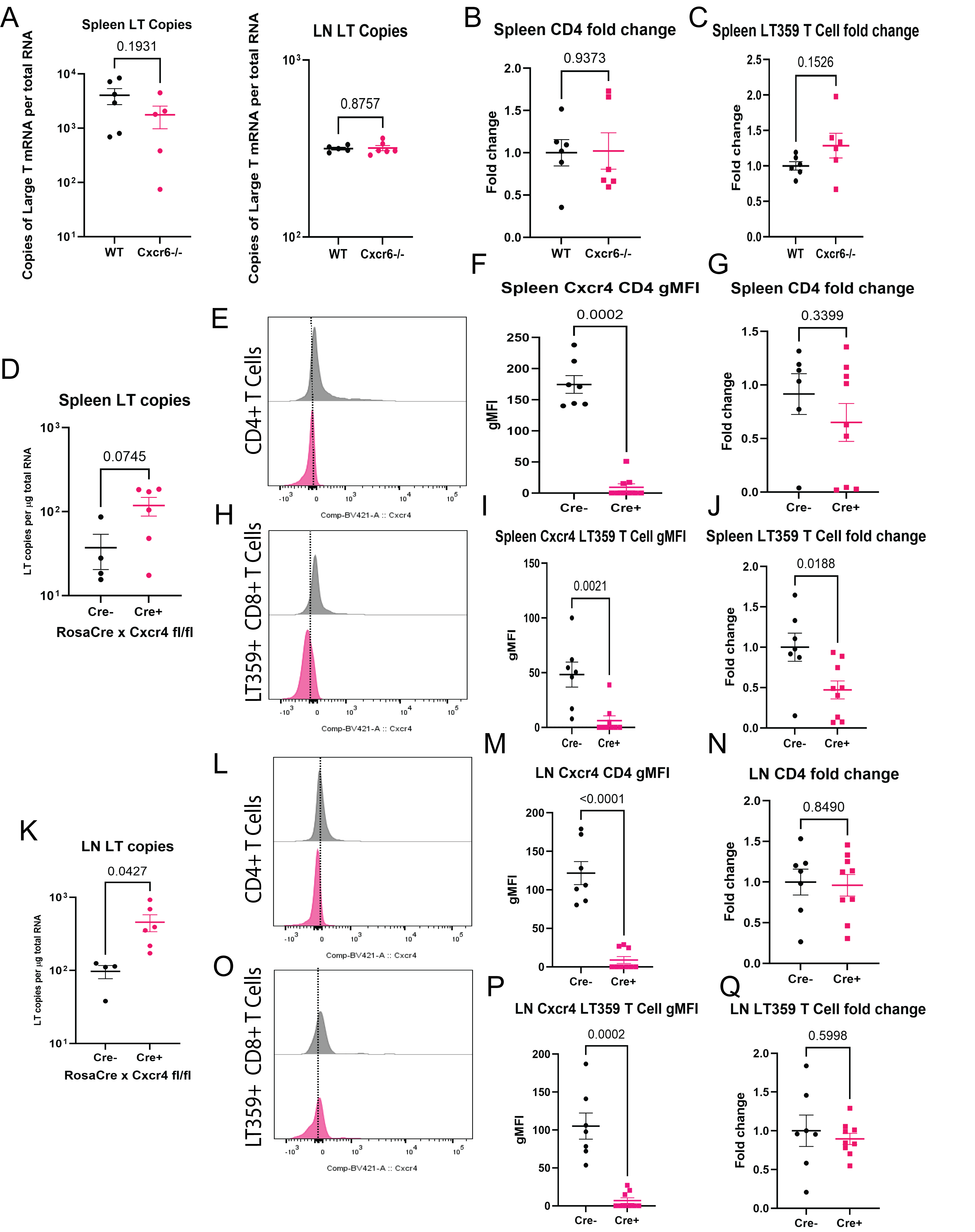

Supplement: Supplementary file 1 — Supplementary Material 1: Supplemental Fig. 1. Brain-specific expression of chemokine receptors on CD4 and CD8 T cells following mupyv infection. WT mice were inoculated i.c. with MuPyV and IV labeled with CD45::FITC prior to euthanasia. Flow cytometry of CCR4, CCR5, CCR6, CCR9, CXCR3 and CXCR5 were assessed. (A) Flow gating strategy. FVD = fixable viability dye. Gating for Cxcr4 + and Cxcr6 + cells were based on fluorescence minus one (FMO) values, and are representative of the gating strategies used for all experiments. FMOs are represented by the dashed line. (B) Representative contour plots depicting expression of CCR5, CCR6, CCR7, and CXCR3 in brain CD4+ T cells. C-D. Quantification of B for C-C receptors (C) and C-X-C receptors (D). E. Expression of CCR6 and CCR7 was increased in brain Db LT359+ CD8+ T cells. F-G. Quantification of E for C-C receptors (F) and C-X-C receptors (G). Flow cytometry, n = 8 mice. Percent positive values were determined using the FMO values for each chemokine. The p-values shown are matched 2-way ANOVA with post-hoc analysis. [file 12974_2025_3496_MOESM1_ESM.png]

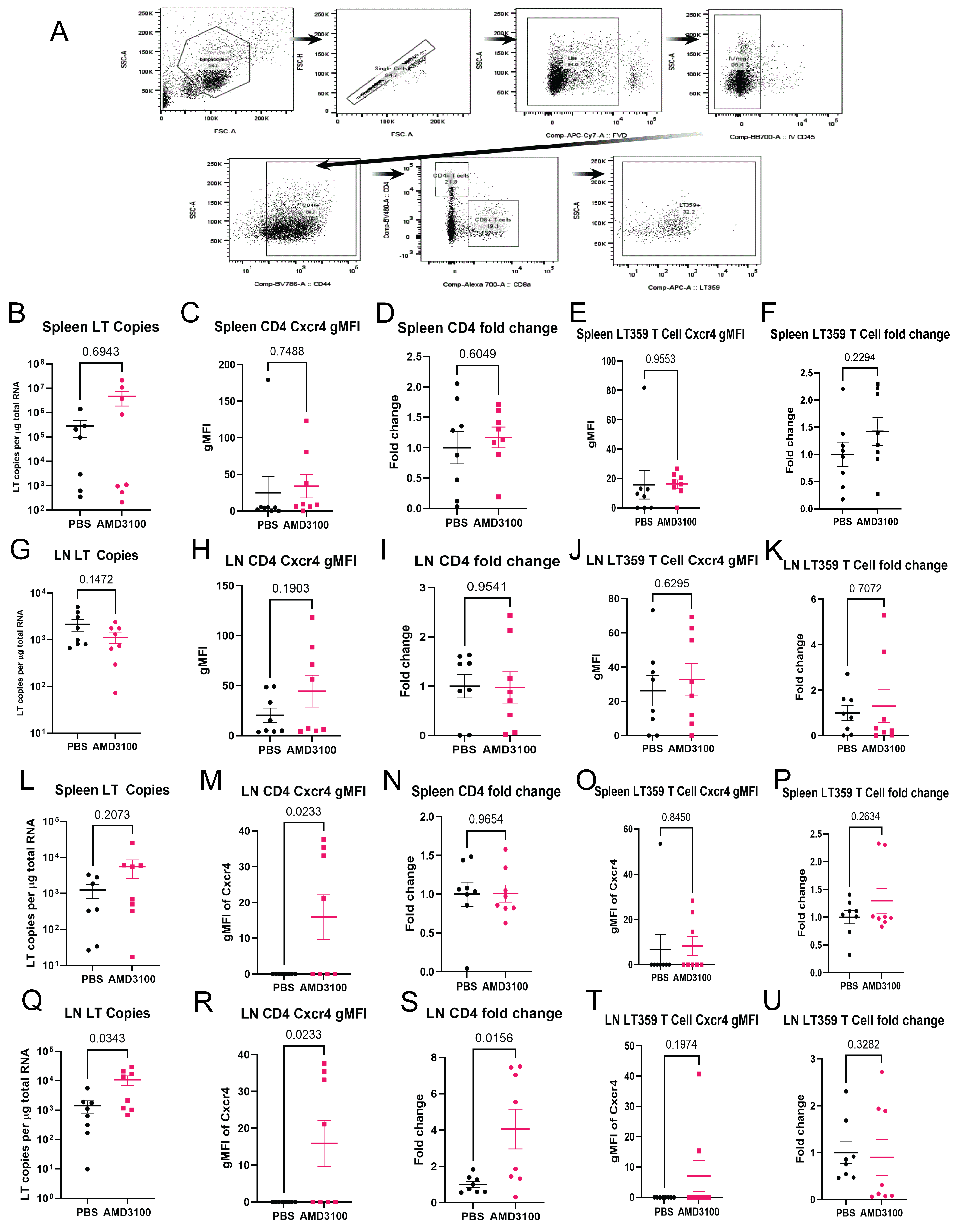

Supplement: Supplementary file 2 — Supplementary Material 2: Supplemental Fig. 2. Loss of either CXCR6 or CXCR4 does not affect Splenic or cervical lymph node T cell numbers after mupyv infection. A-C. WT or CXCR6−/− mice were inoculated i.c. with MuPyV and euthanized at 8 dpi. Tissues were used for flow cytometric analysis of T cell numbers and expression of CXCR4, and qPCR to determine virus levels. n = 6 (WT) or 7 (CXCR6−/−) mice per group, 2 independent experiments. A. LT mRNA copies by qPCR in the spleen and cervical lymph nodes of WT or CXCR6−/− mice. B-C. Number of CD4+ T cells (B) or Db LT359+ CD8+ T cells (C) in the spleens of WT and CXCR6−/− mice calculated as fold change relative to the mean of the WT controls per experiment. D-Q. Rosa-CreERT x Cxcr4fl/fl (Cre+ and Cre−) mice were treated with tamoxifen for 5 d, then i.c. inoculated with MuPyV and euthanized at 8 dpi. Tissues were used for flow cytometry and qPCR. D, K. LT mRNA copies by qPCR in the spleen (D) and cervical lymph nodes (K) of Cre− or Cre+ Rosa-CreERT x Cxcr4fl/fl mice. E, H. Representative histograms of Cre− and Cre+ expression of CXCR4 on CD4+ T cells (E) or Db LT359 tetramer+ CD8+ T cells (H) in the spleen. F, I. gMFI of splenic CXCR4 for CD4+ T cells (F) or Db LT359 tetramer+ CD8+ T cells (I) by Cre expression. G, J. Fold change of the number of CD4+ T cells (G) or Db LT359 tetramer+ CD8+ T cells (J) in the spleen calculated relative to the Cre− controls. L, O. Representative histograms of Cre− and Cre+ expression of CXCR4 on CD4+ T cells (E) or Db LT359 tetramer+ CD8+ T cells (H) in the cervical lymph node. F, I. gMFI of lymph node CXCR4 for CD4+ T cells (F) or Db LT359 tetramer+ CD8+ T cells (I) by Cre expression. G, J. Fold change of the number of CD4+ T cells (G) or Db LT359 tetramer+ CD8+ T cells (J) in the cervical lymph nodes calculated relative to the Cre- controls. n = 6 (Cre−) or 10 (Cre+) mice per group. Data analyzed by a two-tailed Student’s t test. [file 12974_2025_3496_MOESM2_ESM.png]

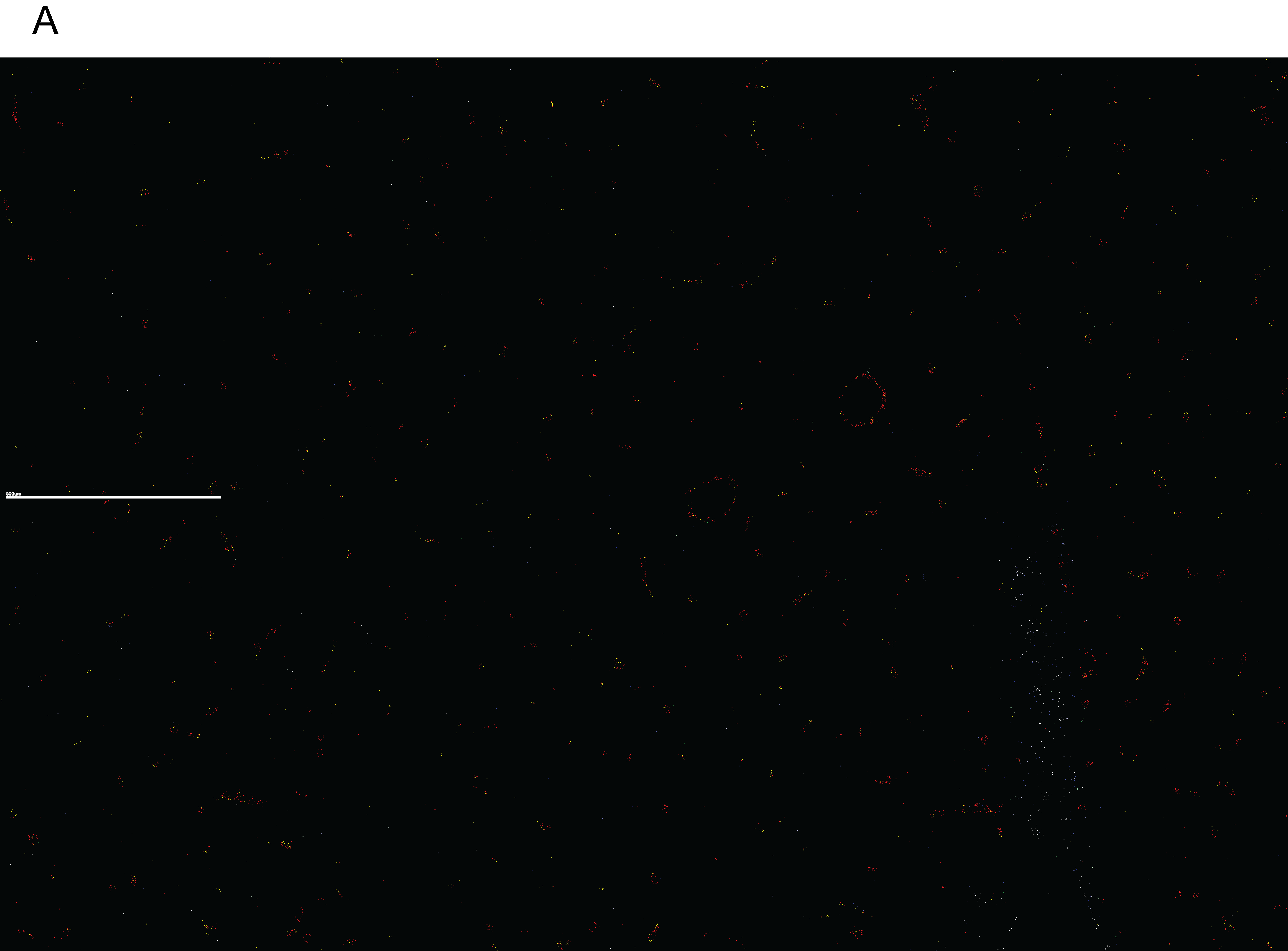

Supplement: Supplementary file 3 — Supplementary Material 3: Supplemental Fig. 3. Inhibition of CXCR4 combined with loss of CXCR6 does not impact spleen or cervical lymph node T cell numbers. WT or CXCR6−/− mice were inoculated i.c. with MuPyV and simultaneously implanted with osmotic pumps filled with either PBS or AMD3100. Mice were weighed daily then euthanized at 8 dpi and tissues were collected for flow cytometry and qPCR. A. Flow cytometry gating strategy. B, G. LT mRNA copies by qPCR in the spleens (B) or cervical lymph nodes (G) of WT mice treated with AMD3100 or PBS. C, H. gMFI of CXCR4 on CD4+ T cells in WT mouse spleens (C) or lymph nodes (H). D, I. Fold change of CD4+ T cells relative to PBS controls in spleens (D) and lymph nodes (I) from WT mice. E, J. gMFI of CXCR4 in virus-specific CD8 + T cells in WT spleens (E) or lymph nodes (J). F, K. Fold change of Db LT359 tetramer+ CD8+ T cells relative to PBS controls in spleens (F) and lymph nodes (K) of WT mice. L, Q. LT mRNA copies by qPCR in the spleens (L) or cervical lymph nodes (Q) of CXCR6−/− mice treated with AMD3100 or PBS. M, R. gMFI of CXCR4 on CD4+ T cells in CXCR6−/− spleens (M) or lymph nodes (R). N, S. Fold change of CD4+ T cells relative to PBS controls in spleens (N) and lymph nodes (S) from CXCR6−/− mice. O, T. gMFI of CXCR4 in Db LT359 tetramer+ CD8+ T cells in CXCR6−/− spleens (O) or lymph nodes (T). P, U. Fold change of Db LT359 tetramer+ CD8+ T cells relative to PBS controls in spleens (P) and lymph nodes (U) of CXCR6−/− mice. n = 8 mice per group, 2 independent experiments. Data analyzed with a two-tailed Student’s t test. [file 12974_2025_3496_MOESM3_ESM.png]

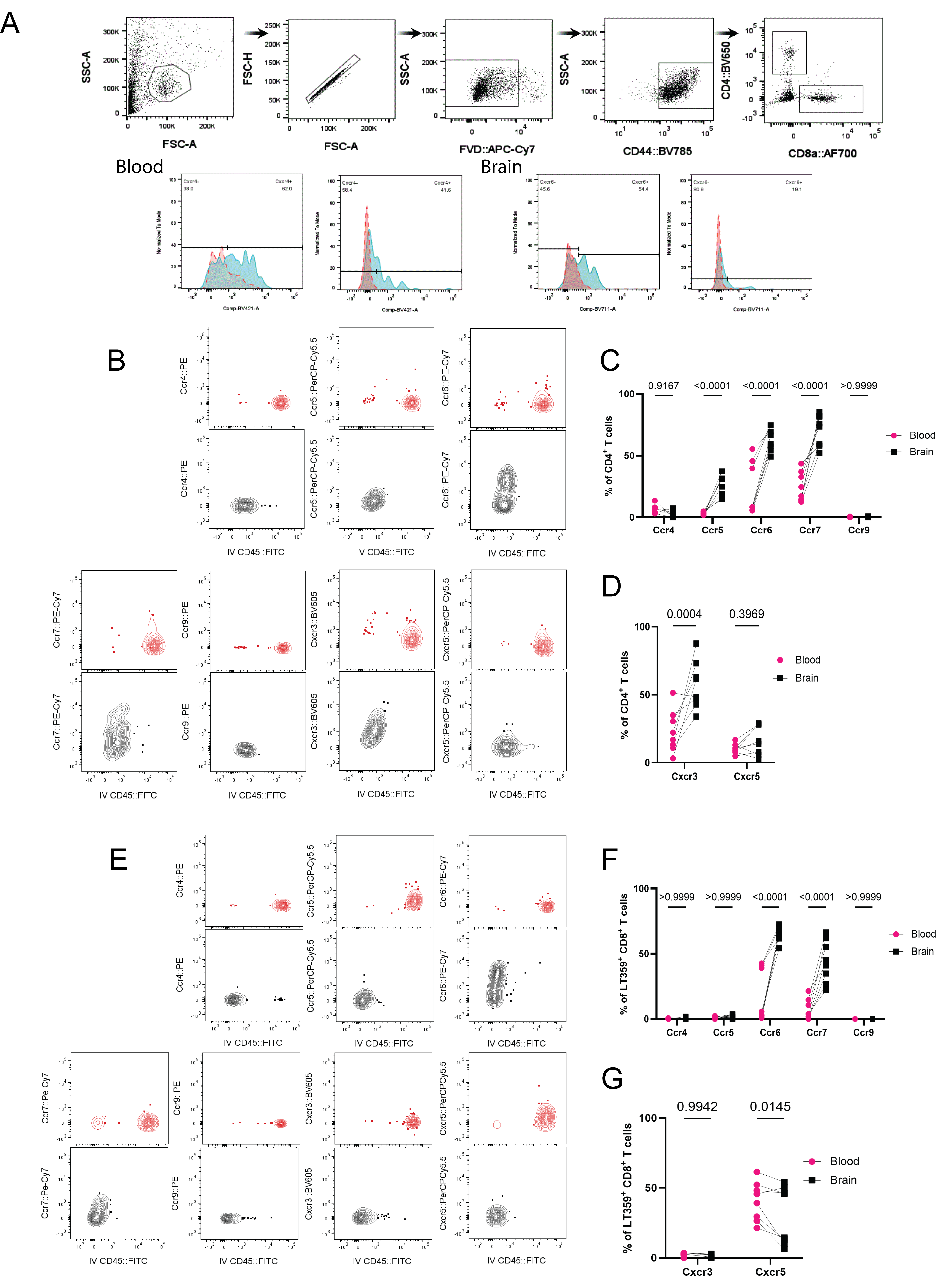

Supplement: Supplementary file 4 — Supplementary Material 4: Supplemental Fig. 4. Spatial transcriptomic analysis shows that CD8+ T cells do not localize with endothelium in brains of MuPyV-infected mice. A. MERFISH transcript visualization shows CD8a (blue), CD4 (green), Cldn5 (red), Flt1 (yellow), and LT antigen (white). [file 12974_2025_3496_MOESM4_ESM.png]
