# Supplementary material for: CXCR4 and CXCR6 dually limit T cell entry into the polyomavirus-infected brain
Source: J Neuroinflammation. 2025 Jun 28;22:169. doi: 10.1186/s12974-025-03496-2 (PMC12205503; doi:10.1186/s12974-025-03496-2)
Supplement: Supplementary file 5 — Supplementary Material 5 [file 12974_2025_3496_MOESM5_ESM.docx]

Supplemental Table 1: Antibodies used for flow cytometry and immunofluorescence.

| **Antibody (clone)::conjugate** | **Dilution** | **Source/RRID** |
| --- | --- | --- |
| ***Flow cytometry*** |  |  |
| TruStain FcX Fc block (93) | 1:200 in 1x PBS | BioLegend/RRID:AB_1574975 |
| *T cell chemokine receptor profiling panel 1* | | |
| CD4 (RM4-5)::BV650 | 1:100 in FACS buffer | BioLegend/RRID:AB_2562529 |
| CD8a (53-6.7)::AF700 | 1:100 in FACS buffer | BioLegend/RRID:AB_493702 |
| CD44 (IM7)::BV786 | 1:100 in FACS buffer | BioLegend/RRID:AB_2571953 |
| CD45 (30-F11)::FITC | 3 ug per mouse in 1x PBS | BioLegend/RRID:AB_312973 |
| LT359::APC | 1:400 in FACS buffer | NIH Tetramer core/RRID:SCR_026557 |
| CCR4 (2G12)::PE | 1:100 in FACS buffer | BioLegend/RRID:AB_1236367 |
| CCR7(4B12)::PE-Cy7 | 1:100 in FACS buffer | BioLegend/RRID:AB_2616688 |
| CXCR4 (L276F12)::BV421 | 1:100 in FACS buffer | BioLegend/RRID:AB_2562788 |
| CXCR5 (2G8)::biotin | 1:100 in FACS buffer | BD Biosciences/RRID:AB_394301 |
| Streptavidin::PerCP-Cy5.5 | 1:200 in FACS buffer | BioLegend/RRID:AB_2868934 |
| CXCR6 (SA051D1)::BV711 | 1:100 in FACS buffer | BioLegend/RRID:AB_2721558 |
| *T cell chemokine receptor profiling panel 2* | | |
| CD4 (RM4-5)::BV650 | 1:100 in FACS buffer | BioLegend/RRID:AB_2562529 |
| CD8a (53-6.7)::AF700 | 1:100 in FACS buffer | BioLegend/RRID:AB_493702 |
| CD44 (IM7)::BV786 | 1:100 in FACS buffer | BioLegend/RRID:AB_2571953 |
| CD45 (30-F11)::FITC | 3 ug per mouse in 1x PBS | BioLegend/RRID:AB_312973 |
| LT359::APC | 1:400 in FACS buffer | NIH Tetramer core/RRID:SCR_026557 |
| CCR5 (HM-CCR5)::biotin | 1:100 in FACS buffer | BioLegend/RRID:AB_313298 |
| Streptavidin::PerCP-Cy5.5 | 1:200 in FACS buffer | BioLegend/RRID:AB_2868934 |
| CCR6 (29-2L17)::PE-Cy7 | 1:100 in FACS buffer | BioLegend/RRID:AB_2072798 |
| CCR9 (9B1)::PE | 1:100 in FACS buffer | BioLegend/RRID:AB_2073249 |
| CXCR3 (CXCR3-173)::BV605 | 1:100 in FACS buffer | BioLegend/RRID:AB_2561353 |
| *Wild-type and Cxcr6^-/-^ T cell counts* | | |
| CD4 (RM4-5)::AF700 | 1:200 in FACS buffer | BioLegend/RRID:AB_493701 |
| CD8a (53-6.7)::PE-Dazzle594 | 1:200 in FACS buffer | BioLegend/RRID:AB_2564026 |
| CD44 (IM7)::BV786 | 1:200 in FACS buffer | BioLegend/RRID:AB_2571953 |
| CD45 (30-F11)::PerCP-Cy5.5 | 3 ug per mouse in 1x PBS | BioLegend/RRID:AB_893344 |
| LT359::APC | 1:400 in FACS buffer | NIH Tetramer core/RRID:SCR_026557 |
| CCR6 (29-2L17)::PE-Cy7 | 1:200 in FACS buffer | BioLegend/RRID:AB_2072798 |
| CXCR4 (L276F12)::BV421 | 1:200 in FACS buffer | BioLegend/RRID:AB_2562788 |
| CXCR6 (SA051D1)::BV711 | 1:200 in FACS buffer | BioLegend/RRID:AB_2721558 |
| *ROSA-Cre x Cxcr4^fl/fl^ T cell counts* | | |
| CD4 (RM4-5)::AF700 | 1:200 in FACS buffer | BioLegend/RRID:AB_493701 |
| CD4 (RM4-5)::BV480 | 1:200 in FACS buffer | BD Biosciences/RRID: AB_2739312 |
| CD8a (53-6.7)::AF700 | 1:200 in FACS buffer | BioLegend/RRID:AB_493702 |
| CD8a (53-6.7)::PE-Dazzle594 | 1:200 in FACS buffer | BioLegend/RRID:AB_2564026 |
| CD44 (IM7)::BV786 | 1:200 in FACS buffer | BioLegend/RRID:AB_2571953 |
| CD45 (30-F11)::FITC | 3 ug per mouse in 1x PBS | BioLegend/RRID:AB_312973 |
| NK1.1 (PK136)::PE | 1:200 in FACS buffer | BioLegend/RRID:AB_313395 |
| NK1.1 (PK136)::biotin | 1:200 in FACS buffer | BioLegend/RRID:AB_313391 |
| Streptavidin::PE-Cy7 | 1:200 in FACS buffer | BD Biosciences/AB_10049577 |
| LT359::APC | 1:400 in FACS buffer | NIH Tetramer core/RRID:SCR_026557 |
| LT359::PE | 1:400 in FACS buffer | NIH Tetramer core/RRID:SCR_026557 |
| CXCR4 (L276F12)::BV421 | 1:200 in FACS buffer | BioLegend/RRID:AB_2562788 |
| CXCR6 (SA051D1)::BV711 | 1:200 in FACS buffer | BioLegend/RRID:AB_2721558 |
| *AMD3100 Cxcr4 inhibition* | | |
| CD3ε (145-2C11)::BV605 | 1:100 in FACS buffer | BioLegend/RRID:AB_2565842 |
| CD3ε (500A2)::PE | 1:200 in FACS buffer | BioLegend/RRID:AB_2629842 |
| CD4 (RM4-5)::BV480 | 1:200 in FACS buffer | BD Biosciences/RRID: AB_2739312 |
| CD8a (53-6.7)::AF700 | 1:200 in FACS buffer | BioLegend/RRID:AB_493702 |
| CD44 (IM7)::BV786 | 1:200 in FACS buffer | BioLegend/RRID:AB_2571953 |
| CD45 (30-F11)::PerCP-Cy5.5 | 3 ug per mouse in 1x PBS | BioLegend/RRID:AB_893344 |
| CD45 (30-F11)::PE | 1:200 in FACS buffer | BioLegend/RRID:AB_31297163 |
| CD45 (30-F11)::BV605 | 1:200 in FACS buffer | BioLegend |
| LT359::APC | 1:400 in FACS buffer | NIH Tetramer core/RRID:SCR_026557 |
| CCR6 (29-2L17)::PE-Cy7 | 1:200 in FACS buffer | BioLegend/RRID:AB_2072798 |
| CXCR4 (L276F12)::BV421 | 1:200 in FACS buffer | BioLegend/RRID:AB_2562788 |
| CXCR6 (SA051D1)::BV711 | 1:200 in FACS buffer | BioLegend/RRID:AB_2721558 |
| ***Immunofluorescence*** | | |
| Mouse CD3ε Rabbit (SP7) | 1:100 in 5% BSA PBST | abcam/RRID:AB_16669 |
| CD8a (4SM16) | 1:100 in 5% Donkey Serum in PBST | eBioscience/RRID:AB_2637159 |
| CD133 | 1:250 in 5% Donkey Serum in PBST | abcam/RRID:AB_19898 |
| CXCR6 Rat Monoclonal IgG (221002) | 1:250 in 5% BSA PBST | R&D Systems/RRID:AB_2277056 |
| Mouse Ki67 Sheep Polyclonal IgG | 1:100 in 5% Donkey Serum in PBST | R&D Systems/RRID:AB_2687500 |
| Human/mouse/rat vimentin goat polyclonal IgG | 1:100 in 5% BSA PBST | R&D Systems/RRID:AB_355153 |
| Rabbit IgG donkey polyclonal IgG::AF488 | 1:250 in 5% BSA PBST | Jackson ImmunoResearch/RRID:AB_2313584 |
| Goat IgG donkey polyclonal IgG::AF555 | 1:250 in 5% BSA PBST | abcam/RRID:AB_2715537 |
| Rat IgG donkey polyclonal IgG::AF556 | 1:250 in 5% BSA PBST | abcam/RRID:AB_2813834 |
| Rat IgG donkey polyclonal IgG::AF647 | 1:250 in 5% BSA PBST | Jackson ImmunoResearch/RRID:AB_2340694 |
| Sheep IgG donkey polyclonal IgG ::AF647 | 1:250 in 5% Donkey Serum in PBST | Jackson ImmunoResearch/RRID:AB_2340751 |

Supplemental Table 2: MERFISH Gene List.

| Gene Input | Vizgen Gene | Ensemble ID | Transcript |
| --- | --- | --- | --- |
| BCL6 | Bcl6 | ENSMUSG00000022508 | ENSMUST00000023151 |
| BSG | Bsg | ENSMUSG00000023175 | ENSMUST00000179781 |
| CCL11 | Ccl11 | ENSMUSG00000020676 | ENSMUST00000000342 |
| CCL2 | Ccl2 | ENSMUSG00000035385 | ENSMUST00000000193 |
| CCL3 | Ccl3 | ENSMUSG00000000982 | ENSMUST00000001008 |
| CCL4 | Ccl4 | ENSMUSG00000018930 | ENSMUST00000019074 |
| CCL5 | Ccl5 | ENSMUSG00000035042 | ENSMUST00000035938 |
| CCR1 | Ccr1 | ENSMUSG00000025804 | ENSMUST00000026911 |
| CCR2 | Ccr2 | ENSMUSG00000049103 | ENSMUST00000055918 |
| CCR3 | Ccr3 | ENSMUSG00000035448 | ENSMUST00000039171 |
| CCR5 | Ccr5 | ENSMUSG00000079227 | ENSMUST00000171579 |
| CCR6 | Ccr6 | ENSMUSG00000040899 | ENSMUST00000231340 |
| CD19 | Cd19 | ENSMUSG00000030724 | ENSMUST00000206325 |
| Cd274 | Cd274 | ENSMUSG00000016496 | ENSMUST00000016640 |
| CD3E | Cd3e | ENSMUSG00000032093 | ENSMUST00000102832 |
| CD4 | Cd4 | ENSMUSG00000023274 | ENSMUST00000024044 |
| CD69 | Cd69 | ENSMUSG00000030156 | ENSMUST00000032259 |
| CD7 | Cd7 | ENSMUSG00000025163 | ENSMUST00000026159 |
| CD79A | Cd79a | ENSMUSG00000003379 | ENSMUST00000003469 |
| CD79B | Cd79b | ENSMUSG00000040592 | ENSMUST00000167143 |
| CD8A | Cd8a | ENSMUSG00000053977 | ENSMUST00000066747 |
| CD8B1 | Cd8b1 | ENSMUSG00000053044 | ENSMUST00000065248 |
| CFAP65 | Cfap65 | ENSMUSG00000047021 | ENSMUST00000094844 |
| CGAS | Cgas | ENSMUSG00000032344 | ENSMUST00000070742 |
| CLDN5 | Cldn5 | ENSMUSG00000041378 | ENSMUST00000043577 |
| CSF1R | Csf1r | ENSMUSG00000024621 | ENSMUST00000025523 |
| CTSD | Ctsd | ENSMUSG00000007891 | ENSMUST00000151120 |
| CTSW | Ctsw | ENSMUSG00000024910 | ENSMUST00000025844 |
| CX3CL1 | Cx3cl1 | ENSMUSG00000031778 | ENSMUST00000034230 |
| CX3CR1 | Cx3cr1 | ENSMUSG00000052336 | ENSMUST00000064165 |
| CXCL1 | Cxcl1 | ENSMUSG00000029380 | ENSMUST00000031327 |
| CXCL10 | Cxcl10 | ENSMUSG00000034855 | ENSMUST00000038816 |
| CXCL11 | Cxcl11 | ENSMUSG00000060183 | ENSMUST00000077820 |
| CXCL12 | Cxcl12 | ENSMUSG00000061353 | ENSMUST00000112866 |
| CXCL13 | Cxcl13 | ENSMUSG00000023078 | ENSMUST00000023840 |
| CXCL16 | Cxcl16 | ENSMUSG00000018920 | ENSMUST00000019064 |
| CXCL2 | Cxcl2 | ENSMUSG00000058427 | ENSMUST00000200681 |
| CXCL5 | Cxcl5 | ENSMUSG00000029371 | ENSMUST00000031318 |
| CXCL9 | Cxcl9 | ENSMUSG00000029417 | ENSMUST00000113093 |
| CXCR1 | Cxcr1 | ENSMUSG00000048480 | ENSMUST00000190313 |
| CXCR2 | Cxcr2 | ENSMUSG00000026180 | ENSMUST00000106899 |
| CXCR3 | Cxcr3 | ENSMUSG00000050232 | ENSMUST00000056614 |
| CXCR4 | Cxcr4 | ENSMUSG00000045382 | ENSMUST00000052172 |
| CXCR5 | Cxcr5 | ENSMUSG00000047880 | ENSMUST00000062215 |
| CXCR6 | Cxcr6 | ENSMUSG00000048521 | ENSMUST00000216072 |
| DTNA | Dtna | ENSMUSG00000024302 | ENSMUST00000115832 |
| EDIL3 | Edil3 | ENSMUSG00000034488 | ENSMUST00000081769 |
| ENSMUSG00000020592 | Sdc1 | ENSMUSG00000020592 | ENSMUST00000020911 |
| ENSMUSG00000024349 | Tmem173 | ENSMUSG00000024349 | ENSMUST00000235495 |
| ENSMUSG00000037894 | H2afz | ENSMUSG00000037894 | ENSMUST00000041045 |
| ENSMUSG00000040296 | Ddx58 | ENSMUSG00000040296 | ENSMUST00000137903 |
| EPN2 | Epn2 | ENSMUSG00000001036 | ENSMUST00000001063 |
| FAM183B | Fam183b | ENSMUSG00000049154 | ENSMUST00000094156 |
| FAS | Fas | ENSMUSG00000024778 | ENSMUST00000025691 |
| FASL | Fasl | ENSMUSG00000000817 | ENSMUST00000000834 |
| FLT1 | Flt1 | ENSMUSG00000029648 | ENSMUST00000031653 |
| FOXJ1 | Foxj1 | ENSMUSG00000034227 | ENSMUST00000036215 |
| FOXP3 | Foxp3 | ENSMUSG00000039521 | ENSMUST00000045566 |
| GABRB1 | Gabrb1 | ENSMUSG00000029212 | ENSMUST00000031122 |
| GM19935 | Gm19935 | ENSMUSG00000110332 | ENSMUST00000209208 |
| GPX1 | Gpx1 | ENSMUSG00000063856 | ENSMUST00000082429 |
| GZMB | Gzmb | ENSMUSG00000015437 | ENSMUST00000015581 |
| H2-AA | H2-Aa | ENSMUSG00000036594 | ENSMUST00000040655 |
| H2-AB1 | H2-Ab1 | ENSMUSG00000073421 | ENSMUST00000040828 |
| HEXB | Hexb | ENSMUSG00000021665 | ENSMUST00000022169 |
| HMGB1 | Hmgb1 | ENSMUSG00000066551 | ENSMUST00000085546 |
| HPGDS | Hpgds | ENSMUSG00000029919 | ENSMUST00000031982 |
| Ifih1 | Ifih1 | ENSMUSG00000026896 | ENSMUST00000028259 |
| IFITM3 | Ifitm3 | ENSMUSG00000025492 | ENSMUST00000026565 |
| IFNA2 | Ifna2 | ENSMUSG00000078354 | ENSMUST00000105147 |
| Ifnar1 | Ifnar1 | ENSMUSG00000022967 | ENSMUST00000023689 |
| Ifnb1 | Ifnb1 | ENSMUSG00000048806 | ENSMUST00000055671 |
| IFNG | Ifng | ENSMUSG00000055170 | ENSMUST00000068592 |
| Ifngr1 | Ifngr1 | ENSMUSG00000020009 | ENSMUST00000020188 |
| IFNLR1 | Ifnlr1 | ENSMUSG00000062157 | ENSMUST00000074408 |
| Il10 | Il10 | ENSMUSG00000016529 | ENSMUST00000016673 |
| IL12B | Il12b | ENSMUSG00000004296 | ENSMUST00000102796 |
| IL12RB2 | Il12rb2 | ENSMUSG00000018341 | ENSMUST00000117441 |
| Il15 | Il15 | ENSMUSG00000031712 | ENSMUST00000209363 |
| Il17a | Il17a | ENSMUSG00000025929 | ENSMUST00000027061 |
| Il1a | Il1a | ENSMUSG00000027399 | ENSMUST00000028882 |
| Il1b | Il1b | ENSMUSG00000027398 | ENSMUST00000028881 |
| Il2 | Il2 | ENSMUSG00000027720 | ENSMUST00000029275 |
| IL23R | Il23r | ENSMUSG00000049093 | ENSMUST00000118364 |
| Il4 | Il4 | ENSMUSG00000000869 | ENSMUST00000127858 |
| Il6 | Il6 | ENSMUSG00000025746 | ENSMUST00000195978 |
| Il7r | Il7r | ENSMUSG00000003882 | ENSMUST00000228782 |
| IRF3 | Irf3 | ENSMUSG00000003184 | ENSMUST00000003284 |
| IRF4 | Irf4 | ENSMUSG00000021356 | ENSMUST00000110307 |
| IRF7 | Irf7 | ENSMUSG00000025498 | ENSMUST00000026571 |
| Itga1 | Itga1 | ENSMUSG00000042284 | ENSMUST00000061673 |
| Itga4 | Itga4 | ENSMUSG00000027009 | ENSMUST00000099972 |
| ITGB5 | Itgb5 | ENSMUSG00000022817 | ENSMUST00000115028 |
| KLRD1 | Klrd1 | ENSMUSG00000030165 | ENSMUST00000112063 |
| KLRK1 | Klrk1 | ENSMUSG00000030149 | ENSMUST00000095412 |
| LGALS3 | Lgals3 | ENSMUSG00000050335 | ENSMUST00000146468 |
| LGMN | Lgmn | ENSMUSG00000021190 | ENSMUST00000110020 |
| LPCAT2 | Lpcat2 | ENSMUSG00000033192 | ENSMUST00000046290 |
| LST1 | Lst1 | ENSMUSG00000073412 | ENSMUST00000097336 |
| LTag MuPyV | LTag MuPyV |  | 2f73dd9f-a139-4378-856c-82e48a542264 |
| LY86 | Ly86 | ENSMUSG00000021423 | ENSMUST00000021860 |
| MAP7 | Map7 | ENSMUSG00000019996 | ENSMUST00000116259 |
| MBP | Mbp | ENSMUSG00000041607 | ENSMUST00000133193 |
| Mki67 | Mki67 | ENSMUSG00000031004 | ENSMUST00000033310 |
| MOBP | Mobp | ENSMUSG00000032517 | ENSMUST00000214943 |
| MS4A6C | Ms4a6c | ENSMUSG00000079419 | ENSMUST00000165310 |
| MYD88 | Myd88 | ENSMUSG00000032508 | ENSMUST00000035092 |
| NCR1 | Ncr1 | ENSMUSG00000062524 | ENSMUST00000006792 |
| NPAS3 | Npas3 | ENSMUSG00000021010 | ENSMUST00000101432 |
| Nr4a1 | Nr4a1 | ENSMUSG00000023034 | ENSMUST00000023779 |
| PCDH15 | Pcdh15 | ENSMUSG00000052613 | ENSMUST00000191854 |
| Pdcd1 | Pdcd1 | ENSMUSG00000026285 | ENSMUST00000027507 |
| PDGFRA | Pdgfra | ENSMUSG00000029231 | ENSMUST00000000476 |
| Prdm1 | Prdm1 | ENSMUSG00000038151 | ENSMUST00000039174 |
| PRF1 | Prf1 | ENSMUSG00000037202 | ENSMUST00000219375 |
| PRR5L | Prr5l | ENSMUSG00000032841 | ENSMUST00000163762 |
| RAN | Ran | ENSMUSG00000029430 | ENSMUST00000031383 |
| RPL6 | Rpl6 | ENSMUSG00000029614 | ENSMUST00000031617 |
| RPL9 | Rpl9 | ENSMUSG00000047215 | ENSMUST00000118543 |
| SIGLECG | Siglecg | ENSMUSG00000030468 | ENSMUST00000005592 |
| SLAMF6 | Slamf6 | ENSMUSG00000015314 | ENSMUST00000194561 |
| SLC4A4 | Slc4a4 | ENSMUSG00000060961 | ENSMUST00000148750 |
| SLCO1C1 | Slco1c1 | ENSMUSG00000030235 | ENSMUST00000205214 |
| SPEF2 | Spef2 | ENSMUSG00000072663 | ENSMUST00000160236 |
| SPOCK2 | Spock2 | ENSMUSG00000058297 | ENSMUST00000121820 |
| STAT1 | Stat1 | ENSMUSG00000026104 | ENSMUST00000186857 |
| STAT2 | Stat2 | ENSMUSG00000040033 | ENSMUST00000085708 |
| TBX21 | Tbx21 | ENSMUSG00000001444 | ENSMUST00000001484 |
| TCF7 | Tcf7 | ENSMUSG00000000782 | ENSMUST00000086844 |
| Tgfb1 | Tgfb1 | ENSMUSG00000002603 | ENSMUST00000002678 |
| TMEM119 | Tmem119 | ENSMUSG00000054675 | ENSMUST00000067853 |
| Tnf | Tnf | ENSMUSG00000024401 | ENSMUST00000167924 |
| TRAC | Trac | ENSMUSG00000076928 | ENSMUST00000198398 |
| TRBC1 | Trbc1 | ENSMUSG00000076490 | ENSMUST00000192856 |
| TREM2 | Trem2 | ENSMUSG00000023992 | ENSMUST00000024791 |
| VCAN | Vcan | ENSMUSG00000021614 | ENSMUST00000109546 |
| VP1 MuPyV | VP1 MuPyV |  | 6dfe2875-de7f-45d7-9e99-02d979b6cf6d |
| VPREB3 | Vpreb3 | ENSMUSG00000000903 | ENSMUST00000000926 |
| WDR17 | Wdr17 | ENSMUSG00000039375 | ENSMUST00000150488 |
| XYLT1 | Xylt1 | ENSMUSG00000030657 | ENSMUST00000032892 |

Supplemental Table 3: Statistics.

| Figure | Analysis | Interaction p/R | Interaction F | n (per group) |
| --- | --- | --- | --- | --- |
| 1I | Two-way RM ANOVA | P=0.8678 | F (1, 8) = 0.02955 | 5,5 |
| 1J | Unpaired Student’s T Test | P=0.0032 | t, df: t=3.461, df=16 | 9,9 |
| 3B | Two-way RM ANOVA | P=0.0319 | F (1, 7) = 7.140 | 6,6 |
| 3C | Two-way RM ANOVA | P=0.0001 | F (1, 7) = 54.71 | 6,6 |
| 3E | Two-way RM ANOVA | P=0.3620 | F (1, 7) = 0.9506 | 6,6 |
| 3F | Two-way RM ANOVA | P<0.0001 | F (1, 7) = 75.49 | 6,6 |
| S1C | Two-way RM ANOVA | P<0.0001 | F (4, 28) = 46.91 | 6,6 |
| S1D | Two-way RM ANOVA | P=0.0043 | F (1, 7) = 17.23 | 6,6 |
| S1F | Two-way RM ANOVA | P<0.0001 | F (4, 28) = 40.56 | 6,6 |
| S1G | Two-way RM ANOVA | P=0.0367 | F (1, 7) = 6.631 | 6,6 |
| 4A | Unpaired Student's t test | 0.8862 | t,df: t=0.1473, df=9 | 5,6 |
| 4B | Unpaired Student's t test | 0.8795 | t,df: t=0.1560, df=9 | 5,6 |
| 4C | Mann-Whitney test | 0.4286 | U=10, rank sum: 35, 31 | 5,6 |
| 4D | Unpaired Student's t test | 0.8674 | t,df: t=0.1723, df=8 | 4,8 |
| 4F | Unpaired Student's t test | 0.024 | t,df: t=2.553, df=13 | 6,9 |
| 4G | Unpaired Student's t test | 0.4717 | t,df: t=0.7396, df=14 | 7,9 |
| 4I | Unpaired Student's t test | 0.3396 | t,df: t=0.9914, df=13 | 6,9 |
| 4J | Unpaired Student's t test | 0.1816 | t,df: t=1.412, df=13 | 6,9 |
| S2A1 | Unpaired Student's t test | 0.1931 | t, df: t=1.407, df=9 | 5,6 |
| S2A2 | Unpaired Student's t test | 0.8757 | t, df: t=0.1609, df=9 | 5,6 |
| S2B | Unpaired Student's t test | 0.9373 | t, df: t=0.08064, df=10 | 6,6 |
| S2C | Unpaired Student's t test | 0.1526 | t, df: t=1.548, df=10 | 6,6 |
| S2D | Unpaired Student's t test | 0.0745 | t, df: t=2.050, df=8 | 4,6 |
| S2F | Mann-Whitney test | 0.0002 | U=0, rank sum: 91, 45 | 7,9 |
| S2G | Unpaired Student's t test | 0.3399 | t, df: t=0.9907, df=13 | 6,9 |
| S2I | Unpaired Student's t test | 0.0021 | t, df: t=3.766, df=14 | 7,9 |
| S2J | Unpaired Student's t test | 0.0188 | t, df: t=2.656, df=14 | 7,9 |
| S2K | Unpaired Student's t test | 0.0427 | t, df: t=2.408, df=8 | 4,6 |
| S4M | Unpaired Student's t test | <0.0001 | t, df: t=7.996, df=14 | 7,9 |
| S2N | Unpaired Student's t test | 0.849 | t, df: t=0.1940, df=14 | 7,9 |
| S2P | Mann-Whitney test | 0.0002 | U=0, rank sum: 91,45 | 7,9 |
| S2Q | Unpaired Student's t test | 0.5998 | t, df: t=0.5368, df=14 | 7,9 |
| 5C | Unpaired t test | P=0.3892 | t=0.8886, df=14 | 8,8 |
| 5D | Unpaired t test | P=0.6389 | t, df: t=0.4796, df:14 | 8,8 |
| 5E | Unpaired t test | P=0.5558 | t, df: t=0.6036, df=14 | 8,8 |
| 5F | Unpaired t test | P=0.1068 | t, df: t=1.724, df=14 | 8,8 |
| 5G | Unpaired t test | P=0.8141 | t, df: t=0.2396, df=14 | 8,8 |
| 5H | Unpaired t test | P=0.3051 | t, df: t=1.064, df=14 | 8,8 |
| 5I | Unpaired t test | P=0.9064 | t, df: t=0.1197, df=14 | 8,8 |
| 5J | Unpaired t test | P=0.0127 | t, df: t=2.856, df=14 | 8,8 |
| 5K | Unpaired t test | P=0.2748 | t, df:t=1.137, df=14 | 8,8 |
| 5L | Unpaired t test | P=0.0095 | t, df: t=3.003, df=14 | 8,8 |
| S3B | Unpaired t test | P=0.6943 | U=24. Sum ranks= 68, 52 | 8,8 |
| S3C | Unpaired t test | P=0.7488 | t, df: t=0.3266, df=14 | 8,8 |
| S3D | Unpaired t test | P=0.6049 | t, df: t=0.5292, df=14 | 8,8 |
| S3E | Unpaired t test | P=0.9553 | t, df: t=0.05706, df=14 | 8,8 |
| S3F | Unpaired t test | P= 0.2294 | t, df: t=1.257, df=14 | 8,8 |
| S3G | Mann Whitney Test | P=0.1472 | t, df: t=1.534, df=14 | 8,8 |
| S3H | Unpaired t test | P=0.1903 | t, df: t=1.376, df=14 | 8,8 |
| S3I | Unpaired t test | P=0.9541 | t, df:t=0.05858, df=14 | 8,8 |
| S3J | Unpaired t test | P= 0.6295 | t, df: t=0.4932, df=14 | 8,8 |
| S3K | Unpaired t test | P=0.7072 | t, df: t=0.3833, df=14 | 8,8 |
| S3L | Unpaired t test | P=0.2073 | t, df= t=1.327, df=13 | 8,8 |
| S3M | Unpaired t test | P=0.0017 | t, df: t=3.859, df=14 | 8,8 |
| S3N | Unpaired t test | P=0.9654 | t, df:t=0.04413, df=14 | 8,8 |
| S3O | Unpaired t test | P=0.8450 | t, df: t=0.1992, df=14 | 8,8 |
| S3P | Unpaired t test | P=0.2634 | t, df: t=1.165, df=14 | 8,8 |
| S3Q | Unpaired t test | P=0.0343 | t, df: t=2.345, df=14 | 8,8 |
| S3R | Unpaired t test | P=0.0233 | t, df: t=2.545, df=14 | 8,8 |
| S3S | Unpaired t test | P=0.0156 | t, df: t=2.750, df=14 | 8,8 |
| S3T | Unpaired t test | P=0.1974 | t, df: t=1.353, df=14 | 8,8 |
| S3U | Mann Whitney Test | P=0.3282 | U= 22. Rank sum: 78,58 | 8,8 |
